# Greedy Ensemble Hyperspectral Anomaly Detection

**DOI:** 10.3390/jimaging10060131

**Published:** 2024-05-28

**Authors:** Mazharul Hossain, Mohammed Younis, Aaron Robinson, Lan Wang, Chrysanthe Preza

**Affiliations:** 1 Computer Science Department, The University of Memphis, Memphis, TN 38152, USA; 2 Electrical and Computer Engineering Department, The University of Memphis, Memphis, TN 38152, USA

**Keywords:** hyperspectral images, anomaly detection, machine learning, stacking ensemble, image processing, remote sensing, statistical methods for HSI, unmanned aerial vehicles, UAV, unmixing, near infrared, NIR

## Abstract

Hyperspectral images include information from a wide range of spectral bands deemed valuable for computer vision applications in various domains such as agriculture, surveillance, and reconnaissance. Anomaly detection in hyperspectral images has proven to be a crucial component of change and abnormality identification, enabling improved decision-making across various applications. These abnormalities/anomalies can be detected using background estimation techniques that do not require the prior knowledge of outliers. However, each hyperspectral anomaly detection (HS-AD) algorithm models the background differently. These different assumptions may fail to consider all the background constraints in various scenarios. We have developed a new approach called Greedy Ensemble Anomaly Detection (GE-AD) to address this shortcoming. It includes a greedy search algorithm to systematically determine the suitable base models from HS-AD algorithms and hyperspectral unmixing for the first stage of a stacking ensemble and employs a supervised classifier in the second stage of a stacking ensemble. It helps researchers with limited knowledge of the suitability of the HS-AD algorithms for the application scenarios to select the best methods automatically. Our evaluation shows that the proposed method achieves a higher average F1-macro score with statistical significance compared to the other individual methods used in the ensemble. This is validated on multiple datasets, including the Airport–Beach–Urban (ABU) dataset, the San Diego dataset, the Salinas dataset, the Hydice Urban dataset, and the Arizona dataset. The evaluation using the airport scenes from the ABU dataset shows that GE-AD achieves a 14.97% higher average F1-macro score than our previous method (HUE-AD), at least 17.19% higher than the individual methods used in the ensemble, and at least 28.53% higher than the other state-of-the-art ensemble anomaly detection algorithms. As using the combination of greedy algorithm and stacking ensemble to automatically select suitable base models and associated weights have not been widely explored in hyperspectral anomaly detection, we believe that our work will expand the knowledge in this research area and contribute to the wider application of this approach.

## 1. Introduction

Anomaly or outlier detection (AD) aims to identify rare items, events, or observations that deviate significantly from most data and do not conform to a well-defined notion of normal behavior. During the last twenty years, developments in hyperspectral sensing have made it possible to record data from several hundred spectral bands, including those beyond the visible range, in a single acquisition [1]. This increased spectral resolution allows for detailed examination of different materials present in the observational scene. Advancement of hyperspectral imaging (HSI) technologies [2] combined with machine learning (ML) recognition techniques [3] promises improved scene characterization in numerous agricultural [4], environmental, and military applications, such as reconnaissance, surveillance, and target acquisition (RSTA) missions [5,6,7]. **Hyperspectral anomaly detection (HS-AD)** [7] aims to find targets in hyperspectral images that deviate spectrally from their surroundings in a scene. *Supervised* anomaly detection solves this problem using data labeled with ground truth. On the other hand, *unsupervised* anomaly detection uses only unlabeled data. However, hyperspectral imagers are expensive, and only a few standard HS datasets, let alone annotated ones, are available [8]. As a result, most researchers are exploring unsupervised HS-AD algorithms [9] that do not require annotated data, such as those based on statistical methods [7].

Researchers regard the Reed–Xiaoli (RX) [10] detector as the benchmark for hyperspectral anomaly detection due to its simple principle, low computational complexity, and relatively good performance. We thoroughly investigated several hyperspectral anomaly detection methods, such as the RX [10], the Kernel-RX Algorithm (KRX) [11], the Gaussian Mixture RX Anomaly Detector (GMRX) [12], the Complementary Subspace Detector (CSD) [13], Cluster-Based Anomaly Detection (CBAD) [14], Fuzzy Cluster-Based Anomaly Detection (FCBAD) [15], HS-AD with Attribute and Edge-Preserving Filters (AED) [16], HS-AD with Kernel iForest (KIFD) [17], and Local Summation Unsupervised Nearest Regularized Subspace with an Outlier Removal Anomaly Detector (LSUNRSORAD) [18]. We found that each method’s performance depends upon specific background characteristic constraints and is effective only in some scenarios. The issue with many statistics-based anomaly detection methods is that they make Gaussian assumptions [19] about the hyperspectral data during the development of the algorithm. A Gaussian assumption means that the background follows a normal distribution and the anomalies tend to be small and less frequently occurring objects in an image [20]. However, real-life scenarios are much more complicated than this. As a result, the hyperspectral images captured in the natural world show strong non-linearity and non-Gaussianity [21], which is why many anomaly detectors fail to identify anomalies.

Researchers use various strategies to address these anomaly detection challenges, such as modeling the background for complex scenes to consider increased background constraints. The most common approach is to use random sampling to create an ensemble of homogeneous outlier detectors. For example, Lu et al. [22] proposed an ensemble using a collaboration representation-based detector (ERCRD). Merrill et al. [23] presented the Unsupervised Ensemble-Kernel Principal Component Analysis (UE-kPCA) using a reconstruction error in the kPCA feature space as an anomaly score. Gurram et al. [24] proposed Sparse Kernel-based Anomaly Detection (SKAD) using an ensemble of a large number of one-class Support Vector Data Description (SVDD) classifiers. Another strategy is to apply homogeneous outlier detectors to newly extracted features to create an ensemble. For example, Yang et al. [25] proposed a voting average ensemble (ERRX MFs) where multiple feature extraction algorithms extracted the features from a hyperspectral image, and the RX detector used those features to create an ensemble. Alternatively, Wang et al. [26] proposed an ensemble of sub-features (SED) by applying heterogeneous algorithms to the data to create new sub-features, and finally a prior-based tensor approximation algorithm (PTA [27]) used these sub-features for anomaly detection. Our empirical study found that ensemble methods using heterogeneous algorithms yielded superior outcomes. In our previous work [28], we proposed an equal-weight voting method called Hyperspectral Unmixing-based Voting Ensemble Anomaly Detector (HUE-AD) that combined four heterogeneous detectors (Abundance, AED, KIFD, and LSUNRSORAD) to identify anomalies. However, we found that selecting these algorithms from many options is tedious and requires individuals with extensive knowledge in this field. We also found that assigning different weights to the algorithms improves the accuracy compared to the straightforward equal weighting used in HUE-AD. Therefore, we sought to develop a method to make the selection of algorithms and weights systematic and efficient.

We propose a stacking ensemble-based approach called **Greedy Ensemble Anomaly Detection (GE-AD)**, which employs multiple heterogeneous HS-AD methods similar to the previous ensemble approach discussed in [26]. However, what differentiates this work from our previous work is the development of a greedy algorithm to methodically determine the best HS-AD methods as inputs to the ensemble, which results in a system that outperforms the individual anomaly detectors. Moreover, a unique input to our greedy algorithm is the abundance map produced by hyperspectral unmixing as described in our previous work [28]. An abundance map characterizes the distribution of endmembers across a hyperspectral image, where each endmember has a unique spectral signature representing the corresponding material in the image. A pure pixel contains the spectral signature from a single endmember. In contrast, the spectral signature of a mixed pixel is a linear combination of multiple endmembers [6]. As the unmixing process mentioned in [28], we used the noise-whitened Harsanyi Farrand Chang [29] to decompose the measured spectra into a collection of endmembers, the N-FINDR [30] algorithm to determine the proportion of each endmember in the pixel, and spectral unmixing algorithm fully constrained least-squares (FCLS) [31] to produce the ***abundance map*** using the identified endmembers. We further use the Spectral Information Divergence (SID) algorithm [32] to narrow the endmembers to those whose spectra are similar to the targets’ reference spectra we seek. We can obtain these reference spectra from datasets like the ECOSTRESS spectral library [33,34] or extract the particular reference spectra from one of the annotated HS images from the same dataset or other datasets. Our greedy algorithm takes these abundance maps and outputs from the HS-AD methods as inputs and chooses the best set of methods based on their combined average F1-macro score [35] in the ensemble.

We trained and evaluated our method on five real-world HS datasets, including the Airport–Beach–Urban (ABU) dataset, the San Diego dataset, the Salinas dataset, the Hydice Urban dataset, and the Arizona dataset. Our evaluation shows that the proposed method achieves a higher average F1-macro score with statistical significance compared to the other individual methods used in the ensemble. In comparison with the state-of-the-art (SOTA) HS-AD methods (discussed in Section 2), our GE-AD method showed better detection accuracy in terms of the achieved F1-macro score. More specifically, the evaluation using the airport scenes from the ABU dataset shows that GE-AD achieved a 14.97% higher average F1-macro score than our previous method (HUE-AD), at least 17.19% higher than the individual methods used in the ensemble, and at least 28.53% higher than the other SOTA ensemble anomaly detection algorithms. The rest of this paper is arranged as follows. Section 2 briefly reviews the traditional and ensemble HS-AD algorithms. Section 3 describes the proposed GE-AD method. In Section 4, we evaluate our method on five real-world datasets to determine its accuracy and efficiency, and Section 5 discusses the results and observations. Finally, we summarize this paper in Section 6.

## 2. Related Work

This section discusses the state-of-the-art HS-AD algorithms and compiles the related algorithms together.

### 2.1. Individual Hyperspectral Anomaly Detection Algorithms

The Reed–Xiaoli (RX) Anomaly Detector algorithm characterizes the hyperspectral image’s background using the hypercube’s mean and covariance [10,36]. RX calculates the Mahalanobis distance between the background and pixel under test. The pixel will be declared an anomaly if the distance exceeds a predefined threshold [37]. Equation (1) below shows the square of the Mahalanobis distance:

(1) $$D^{2}={\left(x-m\right)}^{T}\;\cdot \;C^{-1}\;\cdot \;\left(x-m\right),$$

where x is the observation vector, m is the vector of mean values of each independent variable, and $C^{-1}$ is the inverse covariance matrix of the independent variables. As mentioned above, the RX algorithm is currently the performance comparison benchmark for most anomaly detection algorithms. It has gained popularity by enabling the detection of anomalies without the need for scarcely available labeled data. However, the RX algorithm does not consider the influence of anomaly targets on the background computation. The Kernel-RX Algorithm (KRX) [11] considers HS image data complex and non-linear. It uses the Gaussian Radial Basis Function (RBF) kernel trick to compute the global normal statistics, project them into a linear model in a higher-dimensional feature space, and apply the RX algorithm in that higher-dimensional feature space. Thus, KRX produces better results than the RX algorithm for a complex scene. One of the main limitations of KRX is that it is computationally intensive. If the dimensions of the hyperspectral image are large, then KRX would also require a large amount of memory. The Gaussian Mixture RX Anomaly Detector (GMRX) [12] fits the Gaussian Mixture Model (GMM), assuming the entire image is the background, and assigns pixels to the highest posterior probability mixture component. Then, it applies the RX algorithm to those pixels. However, GMRX fails if it assumes an incorrect number of components.

The Complementary Subspace Detector (CSD) [13] considers HS image data to be linear, assumes that the background and target are complementary subspaces of principal component analysis (PCA), and applies the RX algorithm in those subspaces. PCA is a linear dimensionality reduction technique that uses the data’s singular value decomposition (SVD) to project it to a lower dimensional space.

Cluster-Based Anomaly Detection (CBAD) [14] considers each pixel to be strictly a member of one cluster in k-means clustering. It computes the pixel’s Mahalanobis distance to the component mean. Fuzzy Cluster-Based Anomaly Detection (FCBAD) [15] is a novel extension of the CBAD and GMRX algorithms. It assumes that each pixel can have several possible fuzzy logic membership functions in fuzzy c-means clustering and computes each pixel’s Mahalanobis distance to the component mean. The weakness of any clustering-based outlier detection is that the efficacy largely depends on the clustering method used, and these methods are hard to optimize for outlier detection. The model is not more flexible as it needs to be adjusted according to the distribution characteristics of the different datasets. It fails if it assumes an incorrect number of components. Finally, the application of clustering techniques to large datasets is usually expensive.

Hyperspectral images provide spatial and spectral data about objects of interest. Spatial information can improve the reliability and stability of anomaly detection if the resolution is high enough. For example, HS-AD with Attribute and Edge-Preserving Filters (AED) [16] consists of two stages. This algorithm assumes that anomalies tend to be smaller and possess unique reflectance signatures and that the pixels belonging to the same class would have a high correlation in the spatial domain. First, it utilizes principle component analysis to reduce the data’s dimensionality. Then, a predefined attribute filter is used along with boolean-based fusion to create the initial detection map. The detection map is then refined using edge-preserving filters to reduce the false positive outliers. AED uses a predefined filter, which may not be suitable for various datasets. Moreover, AED assumes that the anomalies must be small objects, making it unsuitable for detecting the large anomalies in a scene.

Du et al. [38] proposed Local Summation Anomaly Detection (LSAD), which utilizes the second-order Mahalanobis distance and a window filter to create the local summation. LSAD represents the correlation between backgrounds via a correlation matrix. The matrix inverse is used during mathematical computation, making the process computationally intensive. Thus, Tan et al. [18] proposed Local Summation Unsupervised Nearest Regularized Subspace with an Outlier Removal Anomaly Detector (LSUNRSORAD) to improve the process by utilizing a linear combination using multiplication and addition. The general idea of the LSUNRSORAD algorithm is the ability to represent background pixels by its neighbors. In addition, LSUNRSORAD uses two thresholds in the outlier removal process to improve the accuracy; one is called the minimum threshold, and the other is called the maximum threshold. If the value of a pixel is higher than the maximum threshold or lower than the minimum threshold, it would be considered an anomaly. The main disadvantage of this algorithm is that it assumes the background obeys a Gaussian distribution. The probability of degraded performance is increased in scenarios where this assumption does not hold.

The isolation forest (iForest) [39] constructs multiple binary trees by random sub-sampling to isolate anomalies. The iForest assumes that in an HS image, a background pixel is hard to isolate from the neighboring pixels, whereas anomalous pixel separation should be relatively straightforward. As a result, in the constructed trees, regular pixels reside in a deeper part of the trees, while anomalous pixels reside in the most shallow parts of the trees. The HS-AD with Kernel iForest (KIFD) [17] algorithm assumes that anomalies are easily distinguishable and do not occur frequently. First, it maps the data into the kernel space in order to reduce scene complexity. It then applies the PCA to choose the most important features. Next, it uses the iForest algorithm constructs the trees, which outputs an initial detection map that is further improved using locally constructed iForest over sliding windows. The depth of a pixel in the tree determines if the pixel is regular or anomalous.

### 2.2. Ensemble Learning

Ensemble learning is a machine learning technique that combines multiple individual ***base models*** or weak learners to create a stronger, more accurate model with better predictions. The individual base models in an ensemble can be different algorithms (heterogeneous) or the same algorithm (homogeneous), and they can be unsupervised or supervised. Note that, even if the base models are the same algorithm, they may be trained on different subsets (random sampling) of the training data. Furthermore, selecting the best parameter values using various approaches, such as cross-validation, i.e., *hyperparameter tuning*, can optimize the individual base model’s performance. The three main classes of ensemble learning methods are ***bagging***, ***stacking***, and ***boosting***. Stacking uses another ML model to aggregate the base models’ predictions, whereas bagging uses voting, averaging, or summation, and boosting uses a sequence of models to correct the predictions of prior models.

**Figure 1:**
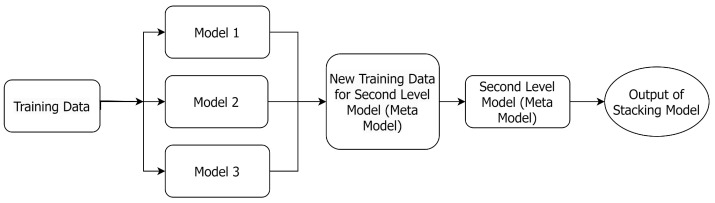
The architecture of a stacked model, where the new training data consist of the outputs of the first-level models.

Figure 1 shows the flowchart of a stacked model [40], which uses output predictions from multiple base models trained on the same dataset to train another ML model (***meta-model***) to make the final predictions [41]. Please note that the meta-model will assign different weights to the base models to maximize the performance of the proposed ensemble method.

### 2.3. Ensemble Learning for Anomaly Detection

Classic hyperspectral anomaly detection algorithms usually make assumptions, including background distributions and the frequency of anomalies. However, real-life scenarios are much more complicated than this, which is why many anomaly detectors fail to identify anomalies. Various techniques are proposed to solve these anomaly detection methods’ instability problems, and ensemble anomaly detection methods are one of them. This section reviews several ensemble learning algorithms related to our work and various approaches to automating the selection and aggregation of the base models for ensemble learning. In Table 1, we summarize all these methods’ similarities and dissimilarities.

The detectors derived from the Collaboration Representation Method (CRD) are classic HS-AD methods. CRD represents each test query as a linear combination of all the training samples from all the classes for image classification [44]. It has a high computational cost due to the use of a dual sliding window to explain a complex scene through a linear combination. Lu et al. [22] proposed an Ensemble and Random CRD (**ERCRD**) method that utilizes random sub-sampling on homogeneous CRD (RCRD) base models to reduce the computational complexity of each RCRD method. It then uses summation to aggregate the detection results from the RCRD methods. Although ERCRD reduces the computational complexity of each RCRD method, the ensemble process still brings its own computational burden without the assurances of complex detection.

Compared to principal component analysis (PCA), kernel PCA (kPCA) can carry out non-linear projections for more complex data. Merrill and Olson [23] proposed Unsupervised Ensemble kPCA (**UE-kPCA**) by suggesting an anomaly scoring function using reconstruction error in kPCA feature space. To reduce computational costs, the authors utilized sub-sampling and an ensemble of homogeneous kPCA base models to refine the final anomalies through averaging. Furthermore, they introduced a novel loss function and automated the kernel parameter selection using batch gradient descent. Despite these advancements, UE-kPCA remains a complex method that requires finding parameters for multiple kPCAs, which can be computationally expensive.

Yang et al. [25] proposed an Ensemble and Random RX with Multiple Features (**ERRX MFs**) anomaly detector. Three features, Gabor, Extended Morphological Profile (EMP), and Extended Multiattribute Profile (EMAP), were computed using heterogeneous base models mixed with the original hyperspectral image (passthrough). They utilized random sub-sampling to compute multiple results for each feature using the RX algorithm. The results for each feature are fused using a voting average ensemble. Then, the four ensembles (for the three features and the original hyperspectral image) produce the final results. This approach avoids the problem of mixing diverse features in an ensemble. However, overrepresented features or noise can dictate the anomaly detection results without standardization.

Wang et al. [26] proposed a subfeature ensemble called **SED**. They removed the noisy bands, normalized the remaining ones, randomly sub-sampled several subfeature sets, and used six different base models in the ensemble. Unlike the ERRX MFs algorithm proposed by Yang et al. [25], SED evaluates the six base models to choose the best-performing methods and uses them to obtain an enhanced feature set. This enhanced feature set is combined with the original hyperspectral image (passthrough) to become the input for the PTA algorithm (meta-model). However, some methods used the same distribution model of the background. Due to background interference, feature redundancy, and noise, the feature enhancement process for these processes may become irrelevant to the final anomaly map. Additionally, SED uses a feature ensemble method rather than an ensemble of different algorithms, as the first-level algorithms aim to obtain the enhanced feature map.

Fatemifar et al. [42] proposed a stacking ensemble for face spoofing anomaly detection that consisted of 63 base classifiers and a Gaussian Mixture Model (GMM) as the meta-classifier for the second stage. The proposed stacking ensemble utilized all 63 classifiers by assigning weights ranging from −8 to 8. While the application for this ensemble is different, it is worth noting that assigning values for each of the 63 weights, even those not performing well, might not result in the most optimized algorithm, especially for the larger dimensional images.

Nalepa et al. [43] proposed a deep learning ensemble for data classification and unmixing that utilized convolutional base models and model augmentation to create new modified models by adding Gaussian noise to the weights of the base models and a second level fuser. The fuser could be as simple as an averaging process, a majority vote, or a supervised second-level fuser. While the proposed method performs well for the tasks of classification and unmixing, it is unclear how effective adding Gaussian noise to the base model weights is when used for detecting anomalous objects.

Ensemble methods yield superior outcomes as they combine the predictions of multiple methods [45]. Aggarwal et al. [46] delved into the effects of using homogeneous weak outlier detectors in the bagging ensemble method in their research. On the other hand, we found that combining heterogeneous weak detectors in an ensemble creates a better understanding of the background and provides better prediction results. Thus, we explored this direction using unsupervised heterogeneous weak detectors in our previous work [28]. However, we found that the process of selecting these algorithms from many options and assigning them weights is not systematic or efficient. In this study, we propose the GE-AD method with greedy search as the solution to the above problem.

### 2.4. Hyperspectral Unmixing-Based Voting Ensemble Anomaly Detector (HUE-AD)

HUE-AD [28] is an equal-weight voting method that combined four detectors (Abundance, AED, KIFD, and LSUNRSORAD) to identify anomalies. The inputs to HUE-AD are binarized results created by thresholding the four base models’ results. Based on the three-sigma rule of thumb, a 99.7th percentile threshold can be used to identify the anomalies in normally distributed data. However, Chebyshev’s inequality [47] suggests that at least 88.8% of cases should fall within properly calculated three-sigma intervals, even for variables that are not normally distributed. We conducted tests to improve the F1-macro score and determined that a 97th percentile threshold would be more appropriate for our purpose.

During our evaluation, we found that systematically assigning weights to inputs improved the accuracy compared to the traditional equal-weight HUE-AD algorithm. We also found that the threshold selection can be a significant source of information loss, resulting in complete system failure in extreme cases. Thus, we developed our model stacking solution called GE-AD to find those weights using a supervised meta-learner and a normalization method to use the complete information generated by the base AD algorithms without introducing bias.

## 3. Materials and Methods

In this section, we discuss our proposed solution to the HS-AD problem.

### 3.1. Greedy Ensemble Hyperspectral Anomaly Detector (GE-AD)

We previously proposed HUE-AD [28], an equal-weight voting ensemble of four heterogeneous detectors. Our empirical analysis showed that a weighted voting approach outperforms HUE-AD. However, determining the voting system’s optimal weights requires a training dataset. Drawing inspiration from Feature Weighted Linear Stacking (FWLS) [48,49], we developed a two-stage weighted stacking ensemble with unsupervised weak HS-AD methods in the first stage and a supervised machine learning (ML) method in the second stage to compute the weights. For the first stage, as there are limited annotated hyperspectral image datasets, a supervised ML model may suffer from under-fitting, so we opted to use unsupervised HS-AD methods. For the second stage, as learning the appropriate weights to assign to multiple inputs requires much less training data than learning features from images, we can utilize a supervised ML method. Initially, we explored Logistic Regression [50] and decision trees [51] to determine the weights in the second stage. Ultimately, we opted for random forest [52] due to its promising results. A random forest is a meta-estimator that fits several decision tree classifiers on various sub-samples of the dataset. These sub-samples are generated by randomly drawing from the dataset with replacement. It uses averaging over the predictions from the decision trees to improve the predictive accuracy and control over-fitting.

**Figure 2:**
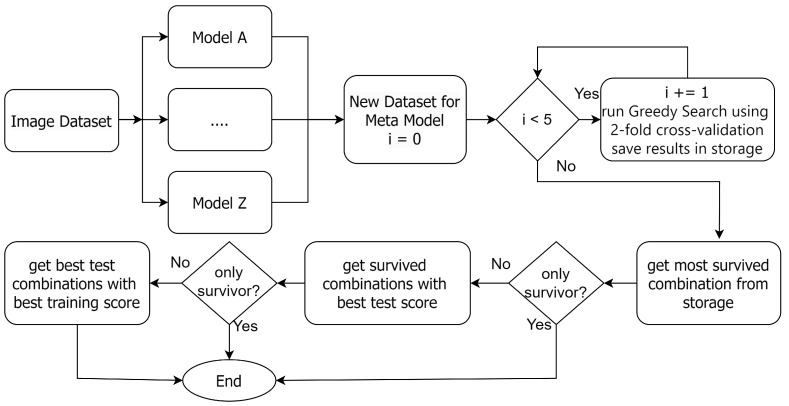
Overall flowchart of GE-AD to find the best AD methods for ensemble fusion.

**Figure 3:**
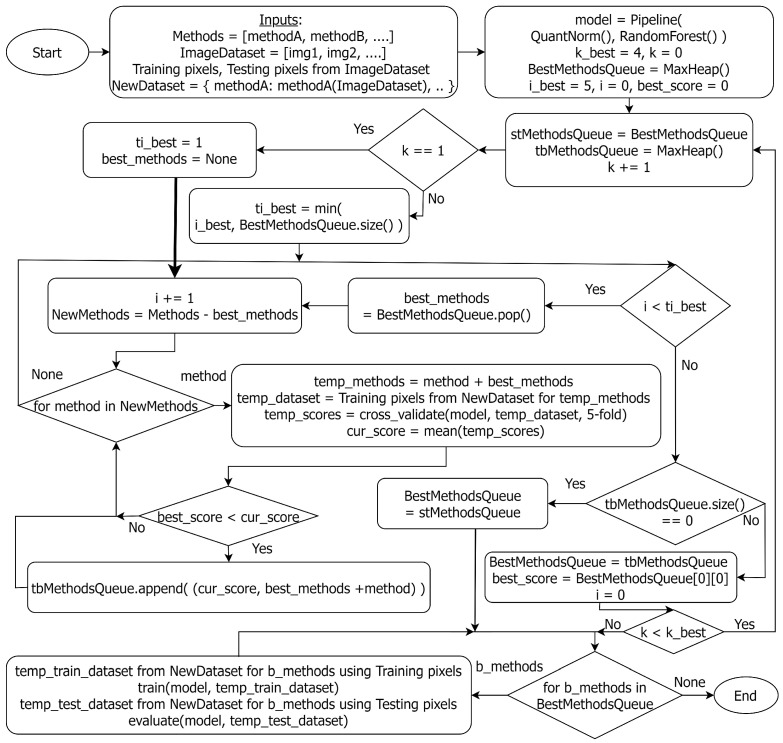
Flowchart of the greedy search to find the best AD methods for ensemble fusion.

Additionally, our empirical analysis showed that the combination of base models that yields the best outcomes varies based on the given scenario. As a result, we put our effort into identifying a more systematic and efficient method for base model selection in a weighted voting ensemble. Figure 2 shows the overall flowchart of the GE-AD algorithm, and our proposed greedy search is depicted in Figure 3. The GE-AD algorithm uses consistent stratified 2-fold cross-validation. *K*-fold cross-validation is a widely used technique to assess the accuracy of a model [53,54,55]. It splits the training dataset into *K* subsets or folds. The model is trained using K−1 of the folds as training data, and the resulting model is validated on the remaining part of the data. The model is trained and evaluated *K* times, with each fold serving as the validation set in turn and being used for validation exactly once. The performance metrics obtained from each fold are then averaged to estimate the model’s generalization performance. In our approach, 2-fold cross-validation divides the pixels in our data into 50–50 split training and testing datasets and evaluates the performance of our GE-AD algorithm twice. Our evaluation found that getting stuck in a local maxima can hinder searching for the best methods. We solved this issue by running the search algorithm five times. We included this 5×2-fold cross-validation as a part of the GE-AD algorithm to ensure that we find the best combinations. Out of these ten sets of results, GE-AD identifies which combinations survived the most. If there is a tie, it uses first the average test score and then the average training score to break the tie.

For our ensemble fusion, we have considered one hyperspectral unmixing algorithm, FCLS, to generate an abundance map and nine AD algorithms, including AED, CSD, FCBAD, GMRX, KIFD, KRX, LSUNRSORAD, Median AD, and RX. In Figure 3, the k_best variable defines the highest number of base models to be used in combination. We varied the k_best variable to determine how many AD methods to include in the ensemble. We found that using four AD methods provides the best results within the constraints of computation complexity. Thus, we set four as the value for k_best. We first applied the AD algorithms to the image data to get each method’s raw anomaly detection output. Then, we used these detection outputs as input features to train the ML model pipeline, which contains quantile normalization and random forest. The greedy search uses 5-fold cross-validation to compute the average F1-macro score of the trained ML model which fuses the output from the selected methods and stores the scores in a Priority Queue (Max Heap). Initially, the top i_best AD methods are selected individually from the priority queue. We varied the i_best variable to determine how many combinations to pick up from the priority queue. We found that using the top five unique performers is enough to provide better results. Thus, we set i_best to be five. Then, in each subsequent round, one more method is added to the set found in the previous iteration. The methods (or combinations of them) that perform better than the best score of the last round are stored again in the queue, and iteration continues. As the number of methods reaches k_best or no better result is found, the greedy algorithm stops its search. We then train the ML pipeline on the training dataset and evaluate the performance using the testing dataset (both datasets are created from the selected methods’ outputs). As the GE-AD model trained on one dataset may differ from that trained on other datasets, we may identify each of these distinct GE-AD models by a different name in Section 4.

Now, we analyze the time complexity of the greedy search algorithm. The greedy search starts with finding the performance of the entire set of *n* AD methods. The priority queue helps greedily choose only the best-performing combinations. Because ML model training time is orders of magnitude higher than the priority queue’s enqueuing and dequeuing time, we do not consider the latter in the complexity analysis. Thus, the runtime of the first round is O(n) as *n* AD methods are evaluated. In the second round, $i_{best}$ best-performing methods were dequeued, each combined with one of the remaining (n − 1) methods for training and evaluation. Thus, the runtime of the second round is $O(i_{best}\times \left(n-1\right))$ for model training computation as up to $i_{best}\times \left(n-1\right)$ AD methods are evaluated and enqueued into the priority queue. Then, in the third round, the $i_{best}$ best-performing combinations of two methods are dequeued, each combined with one of the remaining (n − 2) methods for training and evaluation. A table with all combinations is kept for time optimization and to avoid recomputation. Eventually, $k_{best}$ number of methods are chosen. Then, $n+i_{best}\times \left(n-1\right)+\ldots +i_{best}\times \left(n-k_{best}+1\right)$ training and evaluations are performed resulting in time complexity of

$$O(n+i_{best}\times \left(n-1\right)+i_{best}\times \left(n-2\right)+\ldots +i_{best}\times \left(n-k_{best}+1\right))$$

$$\mathrm{or},O(n),\;\mathrm{when}\;i_{best}\;\mathrm{and}\;k_{best}\;\mathrm{are}\;\mathrm{small}\;\mathrm{constants}.$$

On the other hand, a brute-force search trains and evaluates $C\left(n,1\right)+C\left(n,2\right)+\ldots +C\left(n,k_{best}\right)$ combinations, with a time complexity of

$$O(C\left(n,1\right)+C\left(n,2\right)+\ldots +C\left(n,k_{best}\right))$$

$$\mathrm{or},\;O(C\left(n,k_{best}\right))=O(\frac{n!}{\left(n-k_{best}\right)!k_{best}!})∼O(n^{k_{best}}),\;\mathrm{when}\;k_{best}\;\mathrm{is}\;a\;\mathrm{small}\;\mathrm{constant}.$$

In Section 4, we report the training time for the GE-AD algorithm with random forest as the meta-model.

### 3.2. Datasets

We assessed the AD algorithms’ effectiveness using the scenes from the Airport–Beach–Urban (ABU) dataset [16,56], the San Diego dataset [57,58], the Salinas dataset [59], the Hydice Urban dataset [60], and the Arizona dataset [61].

We used four airport scenes from the ABU dataset [16,56] with dimensions of 100 × 100 pixels, manually extracted from larger images obtained from the Airborne Visible/Infrared Imaging Spectrometer (AVIRIS) website [62]. The AVIRIS is a unique optical sensor that delivers spectral radiance in 224 contiguous spectral channels (bands) with wavelengths from 370 to 2510 nm. The authors removed the noisy bands in the original images using a noise level estimation method [63]. The four images have different spatial resolutions due to variations in flight heights. ABU-I, II, and III have a spatial resolution of 7.1 m per pixel. In comparison, ABU-IV has a spatial resolution of 3.4 m per pixel. The main background includes roofs, shadows, and aircraft parking aprons; airplanes in the image are considered anomaly targets.

The San Diego airport dataset is also a subset of the AVIRIS dataset. It was preprocessed in a previous study [57,58] as follows. The low-SNR (signal-to-noise ratio) and water vapor absorption bands (1–6, 33–35, 97, 107–113, 153–166, and 221–224) were eliminated, and the remaining 189 bands were used. The dataset has two images with a size of 100 × 100 pixels and a spatial resolution of 3.5 m per pixel. The main background is similar to the ABU dataset, and airplanes in the image are considered anomaly targets.

The Salinas dataset [59] is also a subset of the AVIRIS dataset. The dataset was collected by an AVIRIS sensor with 224 channels with a high spatial resolution of 3.7-m pixels and dimensions of 512 × 217 pixels. The 20 removed water absorption bands included 108–112, 154–167, and 224.

The Hydice dataset is a subset of the Urban dataset originating at the US AG center [64]. The dataset was collected by a sensor [65] with a spectral resolution of 10 nm, capturing 162 bands that cover wavelengths ranging from 400 to 2500 nm. The dataset has dimensions of 80 × 100 pixels. The preprocessing done to the dataset included the removal of bands due to atmospheric effects and dense water vapor. The discarded bands included the following bands: 1–4, 76, 87, 101–111, 136–153, and 198–210.

The Arizona dataset, introduced by Watson et al. [61], features a staged scene at the Santa Rita Experimental Range imaged in December 2022. The scene was captured by an unmanned aerial vehicle (UAV) that carried a Pika L sensor manufactured by Resonon [66]. This dataset from Pika L has 281 spectral bands ranging from 400 nm to 1000 nm in wavelength and a spatial resolution of 0.1 m per pixel. The UAV captured the scene from an altitude of 40 m and a speed of 10 m/s. The dataset contains five image scenes of varying sizes from 358 × 293 to 849 × 291. The main background includes soil, vegetation, and water. Vehicles, plastic inflatables, tarps, and miscellaneous foreign objects are considered anomaly targets in this dataset.

### 3.3. Evaluation Metrics

Traditional classification evaluation metrics could be applied to evaluate the proposed GE-AD algorithm. An example of those metrics is the ROC (receiver operating characteristic) curve. It visually represents a binary classifier model’s efficacy across various classification thresholds. The ROC curve plots the true positive rate (TPR) versus the false positive rate (FPR) at each threshold. The decrease in the classification threshold classifies more items as positive, which may increase both false positives and true positives. AUC (area under the ROC curve) [67,68,69] measures the total two-dimensional area under the ROC curve, comprehensively evaluating the model’s performance across all potential classification thresholds.

Another example is the F1 score [70], a harmonic mean of precision and recall. The equations for precision and recall are shown below:

(2) $$Precision=\frac{TP}{TP+FP}$$

(3) $$Recall=\frac{TP}{TP+FN}$$

Here, TP is the number of actual positives predicted as positive, FP is the number of actual negatives predicted as positives, and FN is the number of actual positives predicted as negatives. Because of class imbalance in our dataset (many more negatives than positives), the general F1 score, as shown in Equation (4), will not provide us the perfect insight into the algorithmic performance. With the need for different metrics understood, we used the macro-averaged F1 (or F1-macro) score, as shown in Equation (5). The macro-average calculates the metric independently for each class and then computes the average, treating all classes equally. This approach makes it simpler to demonstrate the impact of improved anomaly detection. Here, *N* is the number of classes. It is computed by taking the mean of all the classes’ F1 scores. In this case, *N* is two as we have two classes.

(4) $$F1\_score=2\times \frac{Precision\times Recall}{Precision+Recall}$$

(5) $$F1\_macro=\frac{\sum_{n=1}^{N}F1\_score_{n}}{N}$$

We note that, when evaluating binary classifiers on *imbalanced* datasets, the false positive rate is less informative than precision in assessing the quality of a classifier [71]. Since anomaly detection typically involves highly imbalanced data (many more negatives than positives), we prefer the F1-macro score over the ROC AUC metric for the evaluation in this paper.

### 3.4. Median-Based Anomaly Detector

RX models the background using the mean value of all the pixels, but the mean is sensitive to outliers. In contrast, the median was previously used for background subtraction in RGB or grayscale images [72]. This motivated us to try the median distance-based method as an outlier detector in HS images and employ the distance between the pixel and the median value as the basis of the evaluation metric. More specifically, we implemented the Median AD method to calculate the absolute difference between the pixel under test and the median value rather than utilizing Mahalnobis distance as the basis of our initial detection map. Then, a threshold using the 97th percentile value was utilized to obtain a binary detection map where each pixel’s value is either 0 (not anomalous) or 1 (anomalous). The main advantages of this method are that it is computationally inexpensive and can work well with only the most informative bands [73] from the hypercube. This effectively eliminates the need to retain all channels processed by the hyperspectral camera for effective and accurate algorithmic performance. This is crucial since HS cubes can be huge files, and band elimination can significantly reduce file sizes. This is extremely important for applications such as drone-based anomaly detection since transferring the complete data cube to a ground station to detect anomalies would be time-consuming and could consume a large portion of finite power resources. The median distance-based method solves this problem because it works well with only a single band/channel. We have used Median AD as an input for our GE-AD algorithm.

The scenes depicted in Figure S1, included in the Supplementary Materials, highlight the performance differences resulting from the implementation of different threshold values for the Median AD method. For example, some targets are missing in the Arizona dataset Image V prediction compared to the ground truth. By increasing the threshold, F1-macro scores improve slightly as the higher threshold removes some false positive detections. Table 2 summarizes the F1-macro score using images from the Arizona dataset thresholded at the 90th, 95th, and 97th percentile values.

### 3.5. Anomaly Detection Results Normalization

We decided to use multiple chosen HS-AD algorithms to improve the accuracy of an ensemble. However, the outputs of the various HS-AD algorithms are often on different scales, incomparable, and not indicative of outlier probabilities [74]. Aggarwal [75], and Zimek et al. [76] investigated outlier detection using ensemble methods and found using multiple methods with wide ranges of output values challenging since this often produces unbalanced ensemble results. Thus, we have investigated potential ways for normalizing anomaly detection scores, such as Välikangas et al. [77], and evaluated various data normalization methods to add to our shortlist of choices.

Zhao et al. [78] used quantile normalization to normalize different types of genes. In our case, we also have diverse output values representing anomalies from different AD algorithms. Quantile normalization can convert these results to a normal distribution without losing meaning. An ML model can learn relations faster from features that follow a normal distribution. In this case, the features are normalized and correlated. The quantile method normalizes the samples by averaging across each quantile row and substituting each point value with the corresponding mean. For example, suppose the four methods we use have the output values 1, 2, 3, and 4 in their corresponding 97th percentile row. Then, since the average of 1, 2, 3 and 4 is 2.5, all the four values in the 97th percentile row will be replaced with 2.5 by the quantile normalization method.

### 3.6. Software Tools and Development

We based our experiment software on the GatorSense Hyperspectral Image Analysis Toolkit [79] as it has multiple AD algorithms implemented in MatLab. All the MatLab code runs on MatLab2022b except the AED algorithm [16], which runs on MatLab2017b. We have also used the Avhyas [80] plugin in QGIS [81] for tasks like SNR computation and spectral unmixing. We implemented our greedy algorithm using Python 3.9. The random forest and all other machine-learning methods are from the Scikit-learn [82] machine-learning library in Python 3.9.

## 4. Results

**Figure 4:**
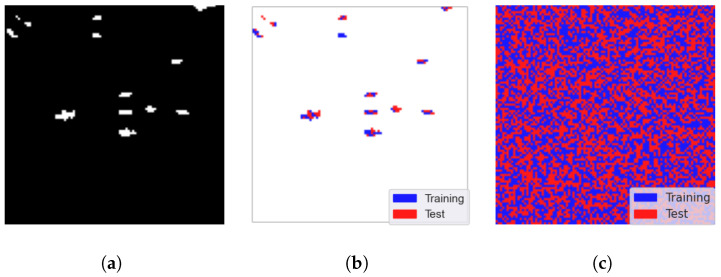
Visualization of the train–test split (seed = 842) of ABU-I data to train our proposed ensemble (GE-AD) method. (**a**) Ground truth, (**b**) split of anomalies only (blue for training and red for testing), and (**c**) complete train–test split.

We evaluate our GE-AD algorithm using consistent stratified 5×2-fold cross-validation. 2-fold cross-validation randomly selects 50% of the pixels as training data and the other 50% for testing. Figure 4b shows the anomalous pixel distribution between the training and testing data from the ABU-IV data. Here, blue denotes the training data, and red denotes the testing data. The actual split is shown in Figure 4c as we used both anomalies and non-anomaly pixels from the image data for training and testing.

The training data has already been used to select the best combination of algorithms. During evaluation, we use the selected algorithms on the testing data to obtain the first-stage anomaly detection output. Next, we run the trained random forest model on the first-stage output to obtain the final anomaly detection output. We repeat this process five times and compute the average F-1 macro scores. All the images are displayed for visualization purposes only.

Table 3 summarizes the training and testing performance results. Specifically, we include the training and testing set detection scores, times required for algorithmic training, and inference times for each dataset. This algorithmic training time includes running the greedy search algorithm to find the best combination and training of the random forest pipeline. It should be noted that the significant increase in time needed to train and run the inference on the Arizona dataset is due to its much higher image resolution than any other dataset included in the comparison. We tested a vanilla brute-force search algorithm on the ABU dataset that took 3001.502 s for comparison and provided the same combination.

We used the one-sided Wilcoxon signed-rank test [83] to compute the statistical significance, which is a modified version of the Mann–Whitney *U* test [84], as we evaluated different algorithms on the same training and testing data. The Mann–Whitney *U* test is a non-parametric counterpart of the *t*-test [85], which provides a more accurate measurement when samples do not follow a normal distribution, even with small sample sizes. We picked the proposed method as a control algorithm and compared its performance with the remaining AD algorithms. The null hypothesis of the Wilcoxon signed-rank test [83] is that the rank sums of the control algorithm **are not significantly less** than those of the other methods. If the *p*-value is much less than the significance level (0.05), the null hypothesis can be rejected, and the result is statistically significant. We ran our tests ten times on eight images from four public datasets (four from ABU, one from Hydice Urban, one from Salinas, and two from San Diego airport). In Table 4, GE-AD has the lowest rank (lower is better), and all the methods have a *p*-value less than 0.05; thus, the null hypothesis can be rejected, and our process is better and differs significantly from the other methods.

### 4.1. Public Dataset Benchmark Evaluation: ABU

**Figure 5:**
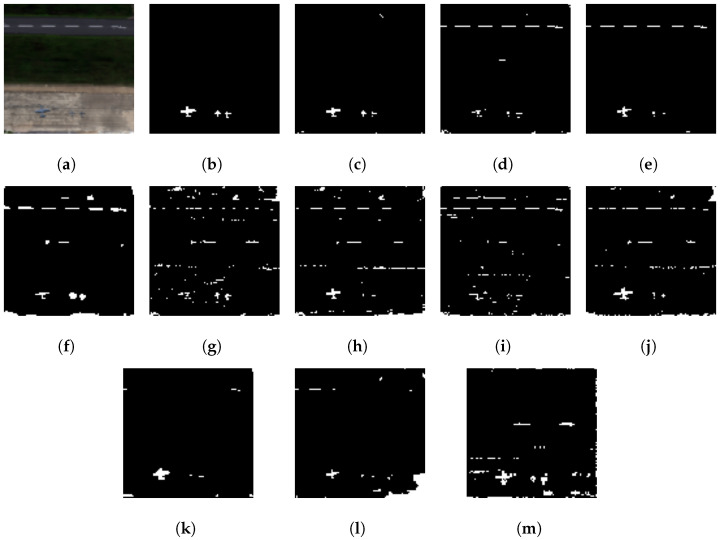
Visual comparison showing better performance of our proposed ensemble method (GE-AD) compared to other methods using ABU-IV data. (**a**) RGB, (**b**) ground truth, (**c**) GE-AD (F1 = 0.856), (**d**) HUE-AD (F1 = 0.649), (**e**) Abundance (F1 = 0.735), (**f**) AED (F1 = 0.610), (**g**) KIFD (F1 = 0.587), (**h**) KRX (F1 = 0.604), (**i**) LSUNRSORAD (F1 = 0.569), (**j**) FCBAD (F1 = 0.631), (**k**) ERRX MF (F1 = 0.666), (**l**) ERCRD (F1 = 0.545), and (**m**) SED (F1 = 0.592). Please note that, as the implementations of algorithms for Figures (**k**–**m**) were not publicly available, we binarized the original outputs from the authors’ papers [22,25,26], respectively.

In this evaluation, the GE-AD algorithm selected the Abundance, AED, FCBAD, and KRX methods as the base models for the ensemble. Subsequently, the individual predictions from these selected algorithms were supplied to the meta-model for the final ML model training and detection. We trained a model with seed 842, which achieved a training F1-macro score of 0.81 and a test F1-macro score of 0.86, a higher F1-macro score than any of the methods used in the ensemble. We are calling this model trained on the ABU dataset GE-AD model-1. GE-AD model-1 produces fewer false positives and creates a clear detection map with all the targets at least partially detected, as shown in Figures S6–S8 and Figure 5 from the ABU dataset.

The null hypothesis of the Wilcoxon signed-rank test [83] is that the rank sums of the control algorithm **are not significantly less** than those of the other base models used in the ensemble. If the *p*-value is less than the significance level (0.05), the null hypothesis can be rejected, and the result is statistically significant. We ran our tests ten times and used all the ABU results to run significance tests. In Table 5, GE-AD has the lowest rank, and all the methods have a *p*-value less than 0.05; thus, the null hypothesis can be rejected, and our process is better and differs significantly from the other methods.

For comparative analysis, we applied the Friedman rank test [86], which measures the significance of the performance differences among the detection methods considered. The null hypothesis of the Friedman rank test is that the rank sums **do not differ** significantly between the three or more “paired” samples, and they are identical. As in the case of the Wilcoxon test, if the *p*-value is less than the significance level (0.05), the null hypothesis can be rejected, and the result is statistically significant. The Friedman test for F1-macro is one-tailed with a *p*-value of 0.05, and ROC AUC is two-tailed [87,88,89] with a *p*-value of 0.05/2. Table 5 shows our method is better and differs significantly from the other methods for the F1-macro metric, with the best average rank of 1.07 (lower is better), and the *p*-value is 3.397^−26^ (numerical zero), which is less than 0.05.

**Figure 6:**
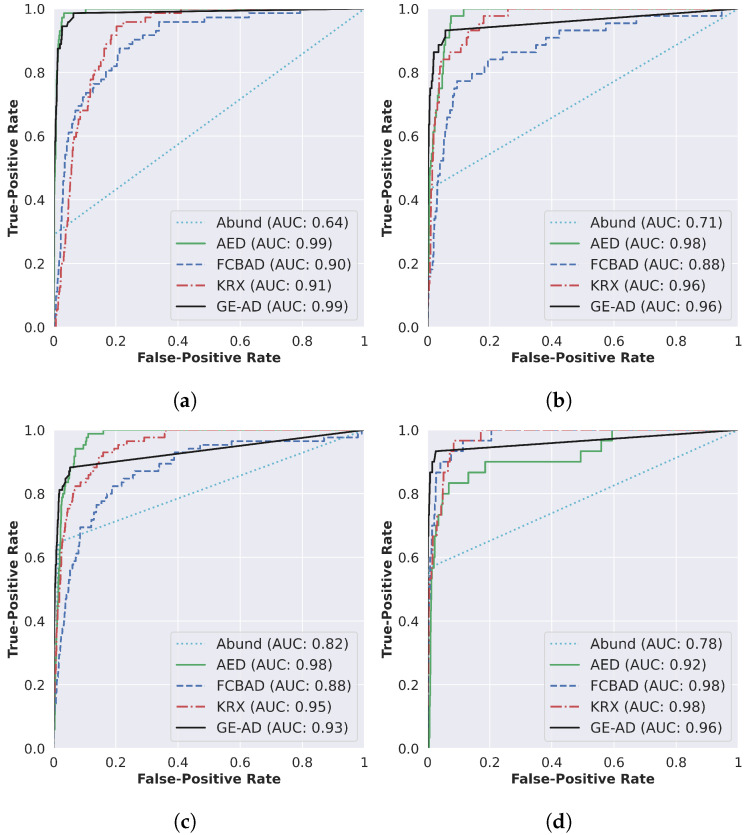
Detection accuracy evaluation through ROC curve and AUC scores (shown in the legend) of (**a**) ABU-I, (**b**) ABU-II, (**c**) ABU-III, and (**d**) ABU-IV.

In addition to the F1-macro metric, we also determined the ROC AUC scores [67] for each algorithm as the authors of other papers evaluated their work using this metric. The results are summarized in Figure 6. Note that even though GE-AD model-1 resulted in outputs that are visually closer to the ground-truth data, it failed to achieve a higher ROC AUC score compared to all the methods used in the ensemble. This demonstrates that a single metric is not sufficient to assess the performance.

As shown in Figure 5 and Figure 6, the proposed method outputs results that are visually closer to the ground-truth data with a lower ROC AUC score; we still conducted the statistical tests on the ROC AUC score and obtained an interesting result. Our evaluation shows that our method is statistically significant for the ROC AUC score over the ten runs. The *p*-value of AED in the one-sided Wilcoxon signed-rank test is 0.038011, much higher than the others but still less than the significance level (0.05). Table 6 shows that the rank of the proposed method is the lowest, and the *p*-value in the Friedman rank test is 3.9^−22^, which is less than 0.05/2.

#### 4.1.1. Comparison with Baseline SOTA Ensemble Methods

We broaden our comparative analysis by elaborating on the performance results of several existing ensemble AD methods. Our GE-AD model-1 struggled to achieve a higher ROC AUC score compared to the other individual methods. Still, it achieved a considerably similar ROC AUC score to the other SOTA ensemble anomaly detection algorithms, as shown in Table 7. However, our previous work [28] discussed how the F1-macro score is a better-suited metric to evaluate anomaly detection tasks as a small number of incorrect or correct predictions can significantly change the AUC score. Additionally, the AUC score does not consider the impact of false positives. Hence, this study has included the ROC AUC score, the F1-macro score, and their corresponding visual comparisons. Our method outperforms the other ensemble methods both visually and in terms of the F1-macro score, as shown in Table 7 and Figure 5, while it provides comparable scores for the ROC AUC.

#### 4.1.2. Generalization Investigation: San Diego Airport

**Figure 7:**
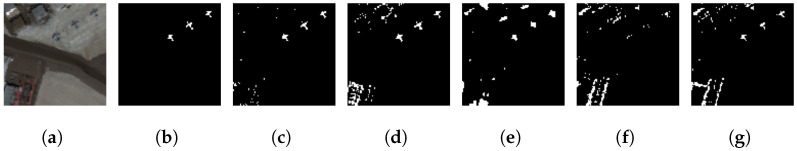
The visualization shows the generalization of our proposed ensemble method (GE-AD) trained on the ABU dataset compared to other methods using San Diego-02 data. (**a**) RGB, (**b**) ground truth, (**c**) GE-AD (F1 = 0.826), (**d**) Abundance (F1 = 0.655), (**e**) AED (F1 = 0.636), (**f**) FCBAD (F1 = 0.536), and (**g**) KRX (F1 = 0.627).

We have also tested our GE-AD model-1 using 100% of the San Diego dataset to evaluate the generalization performance. Figure S9 and Figure 7 show the San Diego Airport dataset results and the visible effects associated with the achieved F1-macro scores of 0.822 and 0.826, respectively. These scores are higher than the input methods used in the ensemble, showing the generalization of our GE-AD model-1.

#### 4.1.3. Ablation Study

**Figure 8:**
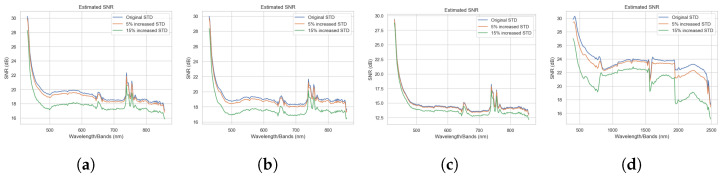
Visualization of SNR values between the original ABU data and the data with the added noise. (**a**) ABU-I, (**b**) ABU-II, (**c**) ABU-III, and (**d**) ABU-IV.

For our first ablation study, we considered the impact of noise on the ensemble. We used the same GE-AD model-1, trained on the original data with no added synthetic noise, as the basis for comparison. The standard deviation (STD) of every channel in the original data was calculated for use as the baseline channel STD metrics. We then computed channel-dependent and normally distributed noise vectors with zero means and STDs that were 5% and 15% greater than the baseline channel STDs. The noise vectors for each channel were then added to their corresponding channel in the original data. This effectively corrupted every channel with additive Gaussian noise and formed two new datasets with 5% and 15% greater STD noise. We can visualize the SNR values between the original ABU dataset and the new noisy dataset in Figure 8, which shows that the ABU dataset already had some noise, and additive Gaussian noise degrades the channel and reduces the SNR.

**Figure 9:**
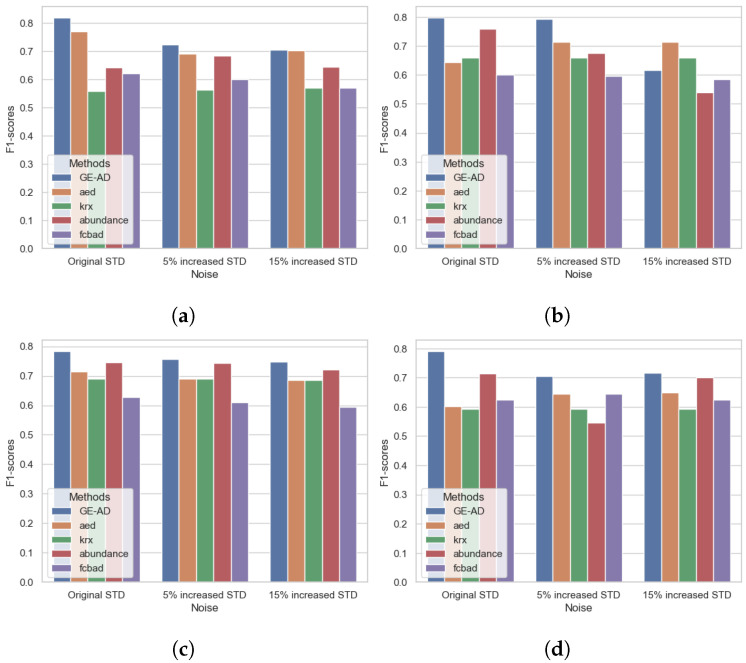
Visualization of F1-macro scores between the original ABU and the data with the added noise. (**a**) ABU-I, (**b**) ABU-II, (**c**) ABU-III, and (**d**) ABU-IV.

**Figure 10:**
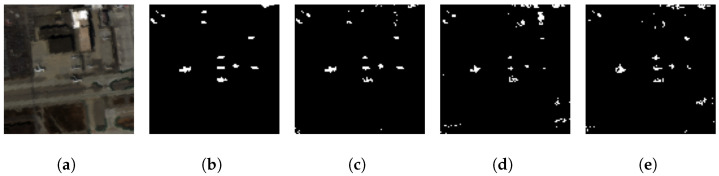
Visual comparison showing qualitative performance of our proposed ensemble method (GE-AD) in the presence of added noise using ABU-I data. (**a**) RGB, (**b**) ground truth, (**c**) GE-AD on original data (F1 = 0.819), (**d**) GE-AD on 5% increased STD data (F1 = 0.725), and (**e**) GE-AD on 15% increased STD data (F1 = 0.706).

In our evaluation, the proposed method performed well under 5% increased STD noise and showed a minor change in the F1-macro score compared to no added noise. However, the method’s performance degraded with the introduction of 15% increased STD noise in the data, as we can see in Figure 9. We can also see that the performance changes in the base models do not follow a pattern. The process depends on these base models and is not exposed to the underlying data. The supervised learning model learns the underlying distribution of the features, and changing that distribution hinders the proposed model’s performance. Still, it performed comparatively well and consistently stayed in the top three. Figure 10 shows some degradation in the results. As Figure 10d shows, three targets were missing, whereas Figure 10e shows that two targets were missing in the detection map. In supplement-III, we have added the table with F1-macro scores and a visualization of the detection maps. In some literature [90,91], noise or corruption is added to training data to improve the ML model, making it more resilient to noise or corruption. As we have not considered this scenario, we have not conducted any data augmentation. The model was not exposed to the understanding of the performance variation in the base models in the presence of noise, distortion, or low SNR. In future work, we will investigate the methodology’s performance improvement with data augmentation.

We also identified the issue of performance variation in the base models when evaluating the Arizona dataset. We had to extensively apply preprocessing before applying the base models to ensure better performance. We have included details in Supplement-I about preprocessing on the Arizona dataset. We applied the same preprocessing for unmixing on the ABU I and III data with 15% greater STD noise on an ad hoc basis and found an improvement in the performance of unmixing, and subsequently improved performance regarding GE-AD. In future work, we will investigate ways to automate the decision to apply preprocessing and apply preprocessing automatically instead of the ad hoc procedure we conduct manually to improve the accuracy.

For our second ablation study, we considered the impact of adding a fourth method and also using all nine methods in the ensemble. Table 8 shows the positive effect of adding the fourth method in the ensemble. Adding FCBAD as the fourth method only decreased the F1-macro score of one of the images.

Table 8 shows that using all nine methods provides better results in two cases. Table 9 shows the individual base model’s runtime. Using all nine methods takes 276.737 s compared to 203.1573 s, the time it takes when using four methods. This longer runtime will be incurred every time we use all nine methods for inference without significant improvement. It shows that limiting the number of base models in stacking helps with the computation time without significant performance degradation. In Table 9, KRX has the longest runtime. Later, we discovered the Fast-KRX [92] method, which we plan to use in the future, as currently, we have not considered individual methods’ time complexity as a metric in the greedy search. So, we have not tested two similar algorithms’ results in our evaluation. We intend to consider time complexity as a metric in a greedy algorithm and use it there in the future.

### 4.2. Public Dataset Benchmark Evaluation: Hydice Urban

**Figure 11:**
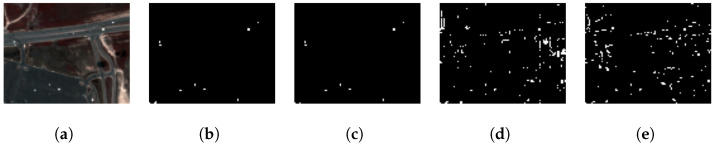
Visual comparison showing better performance of our proposed ensemble method (GE-AD) compared to other methods using HYDICE Urban data. (**a**) RGB, (**b**) ground truth, (**c**) GE-AD (F1 = 0.944), (**d**) FCBAD (F1 = 0.575), and (**e**) LSUNRSORAD (F1 = 0.567).

In this evaluation, the GE-AD algorithm selected the FCBAD and LSUNRSORAD methods as the parallel components for the ensemble. Subsequently, the individual predictions from these selected algorithms were supplied to the meta-model for the final ML model training and detection. We trained a model with seed 577 and achieved a training F1-macro score of 0.81 and a test F1-macro score of 0.94, a higher F1-macro score than any of the methods used in the ensemble. This model produced fewer false positives and created a clear detection map with all the targets at least partially detected, as shown in Figure 11 from the Hydice Urban dataset. We ran our tests ten times using the Hydice Urban dataset and reported the F1-macro scores in Table 10. Our method performed better than any of the methods used in the ensemble.

### 4.3. Public Dataset Benchmark Evaluation: Salinas

In this evaluation, the GE-AD algorithm selected the LSUNRSORAD methods as the only component for the ensemble. Subsequently, the individual predictions from these selected algorithms were supplied to the meta-model for the final ML model training and detection. We trained a model with seed 529 and achieved a training F1-macro score of 0.98 and a test F1-macro score of 0.98, a higher F1-macro score than any of the methods used in the ensemble. This model produced fewer false positives and created a clear detection map with all the targets partially detected, as shown in Figure S10. We ran our tests using the Salinas dataset ten times and reported the F1-macro scores in Table 11. Our method performed better than any of the methods used in the ensemble.

### 4.4. Public Dataset Benchmark Evaluation: San Diego Airport

In this evaluation, the GE-AD algorithm selected the Abundance, GMRX, KIFD, and KRX methods as the parallel components for the ensemble. Subsequently, the individual predictions from these selected algorithms were supplied to the meta-model for the final ML model training and detection. We trained a model with seed 306 and achieved a training F1-macro score of 0.86 and a test F1-macro score of 0.89, a higher F1-macro score than any of the methods used in the ensemble. This model produced fewer false positives and created a clear detection map with all the targets at least partially detected, as shown in Figures S11 and S12. We ran our tests using the San Diego airport dataset ten times and reported the F1-macro scores in Table 12. Our method performed better than any of the methods used in the ensemble.

### 4.5. Private Dataset Benchmark Evaluation: Arizona

The signal-to-noise ratio (SNR) analysis of the hyperspectral images obtained from a new sensor is necessary to ensure the sensor has excellent overall sensitivity and spectral resolution to support capturing and preserving the most critical information in the scene. It increases the efficacy of preprocessing techniques such as geo-rectification [61]. Our project collected the Arizona dataset with a new sensor; therefore, custom preprocessing and initial SNR analyses were required. We applied these preprocessing methods before using them for unmixing and anomaly detection. The details of these preprocessing techniques are discussed in Supplement-I.

**Figure 12:**
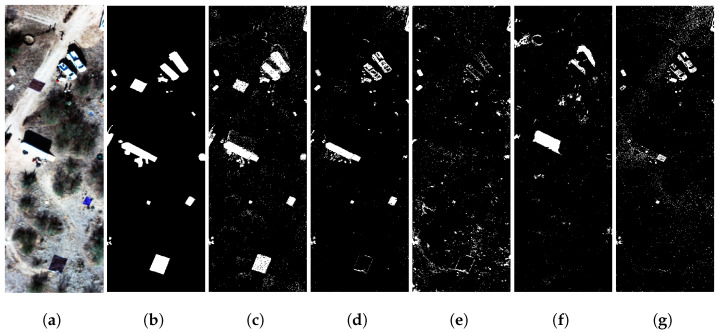
Visual comparison showing better performance of our proposed ensemble method (GE-AD) compared to other methods using Arizona image-III data. (**a**) RGB, (**b**) ground truth, (**c**) GE-AD (F1 = 0.858), (**d**) FCBAD (F1 = 0.773), (**e**) KIFD (F1 = 0.583), (**f**) KRX (F1 = 0.494), and (**g**) LSUNRSORAD (F1 = 0.639).

We trained a separate GE-AD model using the Arizona dataset. In this evaluation, the GE-AD algorithm selected the FCBAD, KIFD, KRX, and LSUNRSORAD methods as the parallel components for the ensemble. Subsequently, the individual predictions from these selected algorithms were supplied to the meta-model for the final ML model training and detection. We trained a model with seed 319 and achieved a training F1-macro score of 0.81 and a test F1-macro score of 0.86, a higher F1-macro score than any of the methods used in the ensemble. This model produced fewer false positives and created a clear detection map with all the targets at least partially detected, as shown in Figure 12 and Figures S13–S16 from the Arizona dataset. We ran our tests using the Arizona dataset ten times, and the comparison between our proposed ensemble and other individual methods using the Arizona dataset is shown in Figure 12 and Table 13. Our method performed better than any of the methods used in the ensemble.

## 5. Discussion

Our GE-AD ensemble anomaly detection method uses prior knowledge to inform the component selection and guide the solution. For example, some ground truth is required to evaluate the algorithmic performance. Utilizing the available information to find a better solution with that prior knowledge is rational. Thus, GE-AD uses the available algorithmic data and greedily selects unsupervised methods to better represent the scenario.

Our GE-AD approach utilizes unsupervised statistical anomaly detection techniques. These techniques leverage the expertise in background information to formulate background characteristics and anomalies that result in more generalized designs. As shown in Table 5 and Table 10, Table 11, Table 12 and Table 13 in Section 4, the outcomes of these weaker techniques differ across various scenarios. However, our ensemble approach was shown to be stable, utilizing information from multiple algorithms and providing consistent generalized results.

In our evaluation, we considered well-known traditional methods that follow basic principles. The performance of these methods is well-established, and the codebase is publicly available. Our method processes the information from these methods and creates a fusion result that outperforms all the input methods. Improved AD methods with higher accuracy may have excellent potential to enhance the performance of GE-AD. As GE-AD outperforms all the input methods in our evaluation, its enhanced results will most likely outperform these high-performing methods. In future work, we will investigate other advanced individual AD methods and their impact on our method’s performance.

We have extensively tested our method on the ABU dataset. Our significance test showed the significance of our process compared to the other included input AD methods. We tested the generalization of the GE-AD model by applying the trained GE-AD model-1 on the San Diego dataset for detection. It achieved F1-macro scores of 0.822 and 0.826 on San Diego images 1 and 2, respectively. As the ABU and San Diego datasets have similar scenes from airports and are collected using similar sensors (AVIRIS), these results demonstrate that the performance of GE-AD is resilient and applicable to similar datasets captured with similar sensors. We conducted an ablation study that showed that GE-AD correctly selects new methods as inputs to improve the performance and maximize the scoring function. In general, our model performed well compared to other baseline SOTA ensembles. However, our method did not perform as well when using ROC AUC as the performance metric as our greedy search algorithm tried to maximize the scoring function, F1-macro, in our search. Based on the F1-macro score, the GE-AD successfully outperformed all the other methods. We note that, based on the specified requirements, the scoring function that the algorithm maximizes can be modified.

Our ablation study with noise showed that our method works well in the presence of low to modest noise corruption as base models can reliably obtain the underlying features, but it is not as robust as we expected it to be. It suffers in the presence of noise when the base models behave differently. Still, it performed comparatively well compared to the other input methods. As we have not considered this scenario, we have not used any data augmentation procedure in our methodology. We think training an ML model with augmented noisy data would have performed differently. Investigating the impact of data augmentation on our greedy algorithm will also be interesting. In future work, we will explore the effect of data augmentation on the methodology’s performance. We will explore how to make the ML model noise-resilient and robust to dataset variation. For the Arizona dataset, we applied preprocessing to address the impact of the noise present in the data. In future work, we will investigate ways to automate this decision to apply preprocessing and apply preprocessing automatically instead of the ad hoc manual procedure to improve the accuracy.

After our evaluation, it is evident that both ROC AUC and F1-macro scores are not adequate metrics when used alone for our requirements. The ROC curve can be skewed when the classes are imbalanced, and we are handling imbalanced data in anomaly detection, where the number of negative examples outweighs the number of positive anomalous examples. The F1-macro can also provide misleading optimism about a system’s performance because of fewer false positives without ensuring that all the targets are identified. In future work, we will investigate a better evaluation metric and scoring function for a greedy search that can acclimate to both dichotomies. We plan to start with Matthews’ correlation coefficient (MCC) [93], which handles situations much closer to ours.

During our evaluation using 2-fold cross-validation, we found that the GE-AD algorithm can become stuck in local maxima and cannot recover. However, applying 2-fold cross-validation multiple times and selecting the combinations that survived in most runs solved this problem. In this paper, we ran the 2-fold cross-validation five times and it produced the expected results. We recommend running the search algorithm at least five times for better, more stable results.

We used an ML model pipeline to combine the quantile normalization step and the random forest model. The ML pipeline is very convenient for training, evaluation, automation, and deployment as it combines the data normalizer and model in one package. However, it has drawbacks, such as the unit tests taking extensive labor to verify the individual steps. We note that the normalization step may be redundant for some ML models. However, we kept the pipeline as we compared multiple different ML models during the selection, and some of them may become susceptible to data variability. Thus, the pipeline standardized our evaluation procedure.

In addition, the GE-AD algorithm can dynamically adjust the AD methods based on their combined performance in the ensemble. When the GE-AD model was trained on the Arizona dataset, our greedy algorithm selected a completely different set of AD methods than the GE-AD model trained using the ABU dataset. The background estimation can change significantly based on the dataset used, and a different set of AD algorithms may be better-suited to describe the new scene. Therefore, our GE-AD method can recognize such changes and produce a new ensemble for a different scenario given that some training data are available.

## 6. Conclusions

We have introduced a new approach called Greedy Ensemble Anomaly Detection (GE-AD) using a supervised ensemble of anomaly detection maps obtained through the hyperspectral anomaly detectors and spectral signature abundance maps obtained through hyperspectral unmixing. The greedy method systematically searches for suitable algorithms to use in the ensemble by maximizing the F1-macro score. It is multiple times faster than the grid search method. Thus, a greedy search helps to compare many algorithms in a shorter period. The random forest ensemble of multiple detection algorithms reduces the instability in obtaining anomalies. It produces better results than the individual HS-AD algorithms. In our investigation, we found ways to reduce the noise in the raw dataset and improve the results of hyperspectral unmixing. We introduced a new Median Anomaly Detector as outliers can distort the statistical characteristics of the RX algorithms. In future work, we plan to create a better stacking ensemble and to exploit the sensor information to create an even more robust GE-AD solution applicable to multiple diverse datasets.

## Figures and Tables

**Table 1:** Feature comparison of ensemble learning algorithms.

Literature	Base Models	Type of Base Models	Base Model Optimization	Base Model Selection	Aggregation
ERCRD [22]	homogeneous	unsupervised	random sampling	N/A	bagging, summation
UE-kPCA [23]	homogeneous	unsupervised	random sampling, gradient descent optimization	N/A	bagging, average
ERRX MFs [25]	heterogeneous, mixed with passthrough	unsupervised	random sampling	not mentioned	bagging, average
SED [26]	only one method or heterogeneous, mixed with passthrough	unsupervised	not mentioned	based on ensemble performance	stacking, unsupervised meta method
Fatemifar et al. [42]	heterogeneous	unsupervised	not mentioned	genetic algorithm	stacking, unsupervised meta method
Nalepa et al. [43]	heterogeneous	supervised	not optimized	not investigated	stacking, supervised meta-learner
HUE-AD [28]	heterogeneous	unsupervised	not optimized	based on individual performance	bagging, voting
GE-AD	heterogeneous	unsupervised	not optimized	greedy search	stacking, supervised meta-learner

**Table 2:** F1-macro score comparison for Median Anomaly Detector using the Arizona dataset (thresholded at different percentile values). Here, the best scores in each scene are shown in bold.

Percentile Values	Image I	Image II	Image III	Image IV	Image V
0.90	0.506	0.534	0.586	0.486	0.595
0.95	0.516	0.562	0.630	0.503	0.643
0.97	**0.519**	**0.575**	**0.650**	**0.513**	**0.670**

**Table 3:** Average training and testing performance in 5×2 runs in terms of F1-macro scores, training, and inference times.

Datasets	Base Models	Training Score	Training Time (s)	Test Score	Inference Time (s)
ABU	Abundance, AED, FCBAD, KRX	0.805±0.011	87.901	0.852±0.086	1.498
Hydice Urban	FCBAD, LSUNRSORAD	0.868±0.063	25.156	0.797±0.048	0.464
Salinas	LSUNRSORAD	0.981±0.018	20.657	0.917±0.108	0.494
San Diego Airport	Abundance, GMRX, KIFD, KRX	0.871±0.008	47.681	0.896±0.050	0.902
Arizona	FCBAD, KIFD, KRX, LSUNRSORAD	0.817±0.002	2983.433	0.805±0.002	24.037

**Table 4:** Performance results of all methods regarding F1-macro metric (%) along with a pairwise Wilcoxon signed-rank test using multiple public datasets.

Method	Avg. F1-Macro Score	Wilcoxon’s *p*-Value [83]
GE-AD	86.452	
Abundance [31]	73.11	3.2748 × 10^−14^
AED [16]	65.282	3.9248 × 10^−15^
FCBAD [15]	59.253	3.9248 × 10^−15^
GMRX [12]	55.453	3.9248 × 10^−15^
KIFD [17]	64.657	3.9244 × 10^−15^
KRX [11]	61.535	3.9248 × 10^−15^
LSUNRSORAD [18]	61.058	3.9248 × 10^−15^

**Table 5:** Comparison of F1-macro scores between our proposed ensemble method (GE-AD), HUE-AD, and individual methods using the ABU dataset.

Method	ABU-I	ABU-II	ABU-III	ABU-IV	Avg. F1 -Macro Score	Wilcoxon’s *p*-Value [83]	Avg. Rank	Friedman’s *p*-Value [86]
GE-AD	0.887	0.878	0.875	0.769	0.852±0.087		1.07	3.397 × 10^−26^
HUE-AD [28]	0.793	0.770	0.751	0.649	0.741±0.058	1.9262 × 10^−8^	2.62	
Abundance [31]	0.670	0.741	0.760	0.736	0.727±0.038	3.033 × 10^−8^	3.10	
AED [16]	0.781	0.646	0.697	0.610	0.683±0.065	1.7847 × 10^−8^	4.40	
FCBAD [15]	0.606	0.603	0.591	0.621	0.605±0.014	1.7847 × 10^−8^	6.75	
KIFD [17]	0.694	0.682	0.695	0.587	0.664±0.046	1.7847 × 10^−8^	5.05	
KRX [11]	0.559	0.658	0.659	0.604	0.620±0.043	1.7847 × 10^−8^	6.42	
LSUNRSORAD [18]	0.695	0.608	0.661	0.568	0.633±0.049	1.7847 × 10^−8^	6.58	

**Table 6:** Performance results of all methods regarding AUC metric (%) along with Friedman rank using the ABU dataset.

Method	ABU-I	ABU-II	ABU-III	ABU-IV	Avg. AUC Score	Wilcoxon’s *p*-Value [83]	Avg. Rank	Friedman’s *p*-Value [86]
GE-AD	0.987	0.980	0.975	0.978	0.980±0.018		2.00	3.901 × 10^−22^
Abundance	0.620	0.733	0.834	0.805	0.748±0.085	1.7847 × 10^−8^	7.15	
AED	0.992	0.980	0.975	0.932	0.970±0.025	3.8011 × 10^−2^	2.48	
FCBAD	0.900	0.896	0.889	0.978	0.916±0.038	1.4139 × 10^−7^	4.28	
KRX	0.914	0.971	0.951	0.975	0.953±0.025	2.7606 × 10^−5^	3.55	

**Table 7:** ABU-IV quantitative performance (as reported in the authors’ papers [22,25,26]) comparison between our proposed ensemble method (GE-AD) and other ensemble methods. Here, the best scores are shown in bold.

	GE-AD	ERRX MF [25]	ERCRD [22]	SED [26]
ROC AUC score	0.9632	0.9970	0.9533	**0.9980**
F1-macro score	**0.856**	0.666	0.545	0.592

**Table 8:** Impact of the new features on our proposed ensemble method (GE-AD) using F1-macro score on the ABU dataset. Here, the best scores in each scene are shown in bold.

	ABU-I	ABU-II	ABU-III	ABU-IV
AED, KRX, Abundance	0.797	0.764	**0.835**	0.754
AED, KRX, Abundance, FCBAD	0.807	0.788	0.784	**0.856**
All nine methods	**0.824**	**0.810**	0.779	0.768

**Table 9:** Comparison of runtime of base AD methods.

Method	Abundance	AED	CSD	FCBAD	GMRX	KIFD	KRX	LSUNRSORAD	RX
Running Time (s)	6.2495	22.8803	0.42967	35.2065	3.8688	48.1669	138.821	20.741	0.37361

**Table 10:** Comparison between our proposed ensemble method (GE-AD) and individual methods using the Hydice Urban dataset.

Method	Avg. F1-Macro Score	Wilcoxon’s *p*-Value [83]	Avg. Rank
GE-AD	0.797±0.048		1.00
FCBAD	0.570±0.012	9.7656 × 10^−4^	2.20
LSUNRSORAD	0.566±0.006	9.7656 × 10^−4^	2.80

**Table 11:** Comparison between our proposed ensemble method (GE-AD) and individual methods using the Salinas dataset.

Method	Avg. F1-Macro Score	Wilcoxon’s *p*-Value [83]	Avg. Rank
GE-AD	0.917±0.108		1.00
LSUNRSORAD	0.548±0.003	9.7656 × 10^−4^	2.00

**Table 12:** Comparison between our proposed ensemble method (GE-AD) and individual methods using the San Diego Airport dataset.

Method	San Diego-01	San Diego-02	Avg. F1 -Macro Score	Wilcoxon’s *p*-Value [83]	Avg. Rank
GE-AD	0.883	0.910	0.896±0.050		1.00
Abundance	0.790	0.655	0.722±0.068	9.5367 × 10^−7^	2.20
GMRX	0.552	0.497	0.524±0.028	9.5367 × 10^−7^	5.00
KIFD	0.774	0.642	0.708±0.067	9.5367 × 10^−7^	2.80
KRX	0.714	0.627	0.671±0.046	9.5367 × 10^−7^	4.00

**Table 13:** Comparison between our proposed ensemble method (GE-AD) and individual methods using the Arizona dataset.

Method	Arizona-I	Arizona-II	Arizona-III	Arizona-IV	Arizona-V	Avg. F1 -Macro Score	Wilcoxon’s *p*-Value [83]	Avg. Rank	Friedman’s *p*-Value [86]
GE-AD	0.770	0.799	0.882	0.816	0.859	0.825±0.047		1.00	3.465 × 10^−29^
FCBAD [15]	0.587	0.614	0.774	0.542	0.812	0.666±0.107	3.7785 × 10^−10^	2.40	
KIFD [17]	0.570	0.569	0.584	0.505	0.578	0.561±0.029	3.7785 × 10^−10^	3.96	
KRX [11]	0.633	0.675	0.494	0.514	0.503	0.564±0.075	3.7785 × 10^−10^	3.40	
LSUNRSORAD [18]	0.520	0.571	0.640	0.498	0.572	0.560±0.049	3.7785 × 10^−10^	4.24	

## Data Availability

The Arizona dataset and our computer code are not publicly available. Their availability is dependent upon the approval from our sponsor. However, our code repository will be made privately available upon proper request to the corresponding author.

## Supplementary Materials

The following supporting information can be downloaded at https://www.mdpi.com/article/10.3390/jimaging10060131/s1, Figure S1: The selection of 0.97 quantile point after applying the Median AD method on Image V from the Arizona dataset; Figure S2. Flowchart for the pre-processing done to improve the F1 score of the abundances; Figure S3. Visual comparison showing qualitative improvement of applying preprocessing; Figure S4. Positive impact of removing noisy bands from Image V in the Arizona dataset; Figure S5. Irregular results from kernel isolation forest anomaly detection are transformed into a normal distribution; Figure S6: Visual comparison showing better performance of our proposed ensemble method (GE-AD) compared to other methods using ABU-I data; Figure S7: Visual comparison showing better performance of our proposed ensemble method (GE-AD) compared to other methods using ABU-II data; Figure S8: Visual comparison showing better performance of our proposed ensemble method (GE-AD) compared to other methods using ABU-III data; Figure S9: The visualization shows the generalization of our proposed ensemble method (GE-AD) trained on the ABU dataset compared to other methods using San Diego-01 data; Figure S10: Visual comparison showing better performance of our proposed ensemble method (GE-AD) compared to other methods using Salinas data; Figure S11:The visualization shows the generalization of our proposed ensemble method (GE-AD) trained on the ABU dataset compared to other methods using San Diego-01 data; Figure S12: The visualization shows the generalization of our proposed ensemble method (GE-AD) trained on the ABU dataset compared to other methods using San Diego-02 data; Figure S13: Visual comparison showing better performance of our proposed ensemble method (GE-AD) compared to other methods using Arizona image-I data; Figure S14: Visual comparison showing better performance of our proposed ensemble method (GE-AD) compared to other methods using Arizona image-II data; Figure S15: Visual comparison showing better performance of our proposed ensemble method (GE-AD) compared to other methods using Arizona image-IV data; Figure S16: Visual comparison showing better performance of our proposed ensemble method (GE-AD) compared to other methods using Arizona image-V data; Figure S17. Visual comparison showing qualitative performance of our proposed ensemble method (GE-AD) in the presence of added noise using ABU-II data; Figure S18. Visual comparison showing qualitative performance of our proposed ensemble method (GE-AD) in the presence of added noise using ABU-III data; Figure S19. Visual comparison showing qualitative performance of our proposed ensemble method (GE-AD) in the presence of added noise using ABU-IV data; Table S1. F1 score comparison for abundances related to anomalies in Arizona Image III; Table S2. F1 score comparison for abundances related to anomalies in Arizona Image V; Table S3. The impact of noise on our proposed ensemble method (GE-AD) using F1-macro on the ABU dataset.
