# Supplementary material for: Greedy Ensemble Hyperspectral Anomaly Detection
Source: J Imaging. 2024 May 28;10(6):131. doi: 10.3390/jimaging10060131 (PMC11204925; doi:10.3390/jimaging10060131)
Supplement: Supplementary file 1 [file jimaging-10-00131-s001.zip › jimaging-2928945-supplementary.pdf]

Supplementary Materials: Greedy Ensemble Hyperspectral Anomaly Detection

Mazharul Hossain [1,†,‡], Mohammed Younis [2,†,‡], Aaron Robinson [2,†], Lan Wang [1,†], and Chrysanthe Preza [2,†*]

1. Supplement I

In this section, we discuss methodology related to our proposed solution to the HS-AD problem when using the experimental Arizona dataset obtained by team members of our collaborative project introduced in Watson et al. [1].

1.1. Median Based Anomaly Detector

The scenes depicted in Figure S1 highlight the performance changes resulting from implementing the improvements mentioned in the main paper. For example, some targets are missing in the Arizona dataset Image V prediction compared to the ground truth. By increasing the threshold, F1 scores improve slightly as the higher threshold removes some false-positive detections.

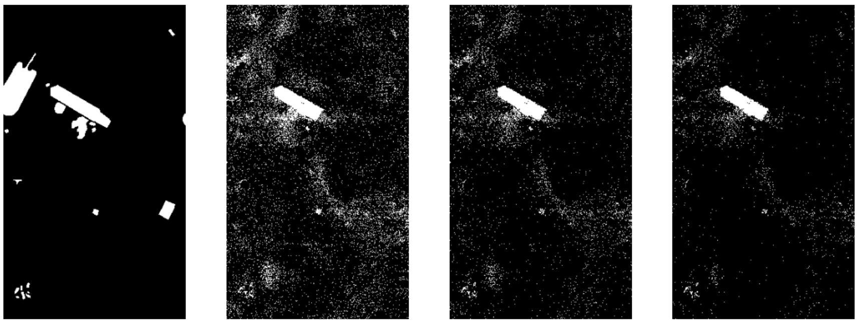

(a) **(b)** **(c)** **(d)**

Figure S1. The selection of 0.97th percentile point after applying the Median AD method on Image V from the Arizona dataset. (a) ground truth for Image V. (b) Median AD method with 0.90th percentile point as a threshold (F1 = 0.595). (c) The Median AD method with 0.95th percentile point as a threshold (F1 = 0.643). (d) The Median AD method with 0.97th percentile point as a threshold (F1 = 0.670).

1.2. Preprocessing Methods to Improve Hyperspectral Unmixing for Arizona Dataset

It is important to point out that hyperspectral sensor selection typically involves a trade-off between its spatial and spectral resolutions. If the sensor has a lower spatial resolution, this will increase the probability of mixed pixels representing the spectral signature of more than one material and, therefore, influence the need for hyperspectral unmixing of the scene [2].

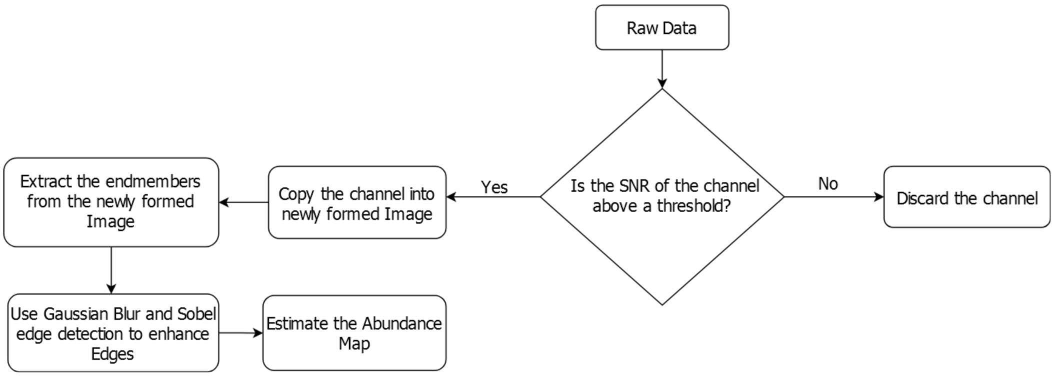

Figure S2. Flowchart for the pre-processing done to improve the F1 score of the abundances.

Multiple image processing techniques were investigated to improve anomaly detection performance using hyperspectral unmixing with low-resolution sensor data to improve the F1 score of the abundances obtained for the anomalies. The first step in the preprocessing is to evaluate the Signal-to-noise ratio (SNR) value and remove noisy bands as described in Section 1.3. We identified the maximum SNR value and kept bands above 97 % of the max SNR or at least 5 dB, whichever is higher. The next step is using Gaussian blur followed by a Sobel edge detector to enhance the edges in the hyperspectral image. This process emphasizes the border of the different objects in the scene. aThe complete process is illustrated in Figure S2. For the unmixing process mentioned in [3], we first used the noise-whitened Harsanyi Farrand Chang [4] algorithm to estimate the number of unique spectra or endmembers. Then, we used the N-FINDR [5] algorithm to estimate those individual spectra. After completing the endmember estimation procedure, we used the fully constrained least-squares (FCLS) [2] method to estimate the abundance map of the unique spectra in the HS image. Finally, we used the spectral similarity information divergence algorithm (SID) [6] to measure the spectral similarity between pixels and specific reference spectra and extract our target spectra as a feature for GE-AD.

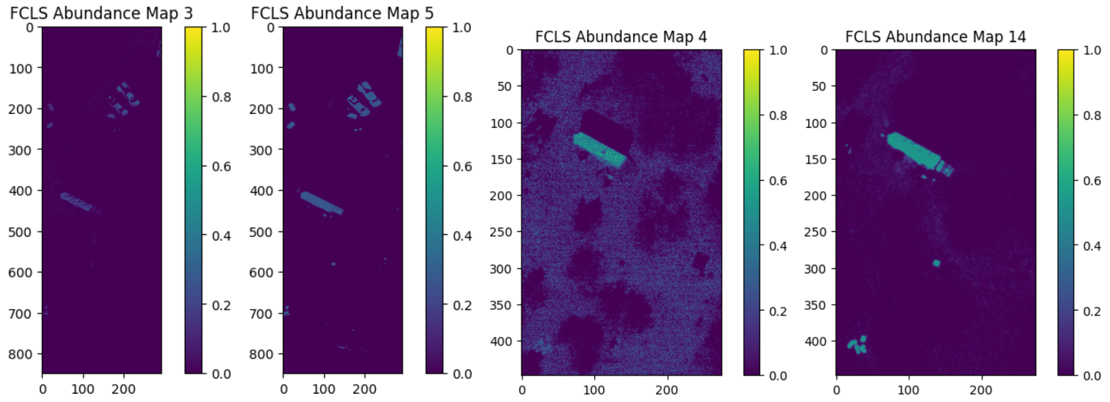

(a) **(b)** **(c)** **(d)**

Figure S3. Visual comparison showing qualitative improvement of applying preprocessing. (a) Abundances for Arizona Image III using an original hyperspectral image and (b) Abundances using the preprocessed hyperspectral image. (c) Abundances for Arizona Image V using an original hyperspectral image and (d) Abundances using the preprocessed hyperspectral image.

Figure S3 qualitatively depicts the improvements resulting from the implementation of the preprocessing techniques as better contrast and less clutter are observed in the imagery as you proceed from figures S3a to S3d. The quantitative improvement of these preprocessing methods on the average F1 score is demonstrated in Tables S1 and S2. Upon checking Tables S1 and S2, it becomes evident that the abundance obtained using preprocessing outperforms the abundances obtained from implementation in Matlab [7] and the original image without any preprocessing. The preprocessing steps were subsequently retained as a permanent component of our algorithmic process since it satisfied the ultimate goal of improving the F1 score of the abundance and anomaly detection ensemble method.

Table S1. F1 score comparison for abundances related to anomalies in Arizona Image III

Method	class 0	class 1	F1-Macro
Original Abundance (no preprocessing)	0.97	0.34	0.66
Preprocessed Image Abundance (noisy bands removal, Gaussian Blur, and Sobel Edge Enhancement)	0.98	0.57	**0.78**
Matlab Select bands endmembers [7]	0.97	0.15	0.56

Please note that our custom preprocessing was only done for the Arizona dataset due to the sensor being noisy toward the initial and the later wavelengths. The impact of our preprocessing pipeline becomes obvious when comparing the F1 scores in Tables S1 & S2, in which we compare the performance with and without applying preprocessing. Please note that we have used public benchmark datasets like the ABU Dataset without applying band selection or preprocessing to ensure that we conduct a fair comparison between our proposed method and other methods.

Table S2. F1 score comparison for abundances related to anomalies in Arizona Image V

Method	class 0	class 1	F1-Macro
Original Abundance (no preprocessing)	0.98	0.34	0.66
Preprocessed Image Abundance (noisy bands removal, Gaussian Blur, and Sobel Edge Enhancement)	0.98	0.46	**0.72**
Matlab Select bands endmembers [7]	0.97	0.11	0.54

1.3. Preprocessing Methods to Improve Hyperspectral AD Algorithms for Arizona Dataset

When dealing with hyperspectral images from a new sensor, it is crucial to analyze the SNR to ensure that the sensor has an excellent overall sensitivity and spectral resolution. This supports capturing and preserving the most important information in the scene and increases the efficacy of pre-processing techniques such as geo-rectification [1]. We have computed the SNR for all bands using the Avhyas plugin [8] in QGIS. Many hyperspectral images contain noisy bands, so further analysis of the SNR is needed. Figure S4a displays the SNR for Image 5 from the Arizona dataset. Analysis of the figure reveals that the hypercube tends to be noisy for input wavelengths close to 400 nm and those higher than 860 nm. This suggested a procedure to remove those noisy wavelengths/bands, evaluate the anomaly detector on the new hypercube containing less noisy wavelengths, and create a new hypercube consisting of the bands with signal-to-noise ratios above our predefined threshold. The results of the noisy band removal are depicted in Figures S4a and S4b. Once all noisy bands are removed, we selected the most informative bands using the method proposed by Du and Yang [7] to reduce the hypercube's dimensionality and improve the processing times without degrading performance.

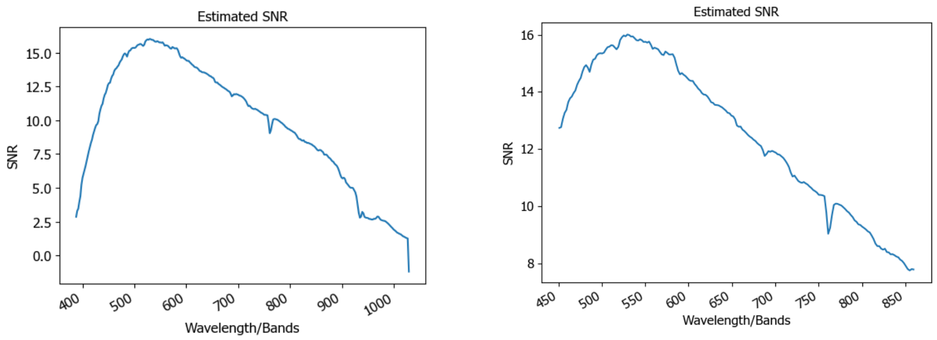

(a) SNR in [dB] for Arizona Image V **(b)** SNR in [dB] after deleting noisy wavelengths

Figure S4. Positive impact of removing noisy bands from Image V in the Arizona dataset.

Analysis of data contained Figures S4a and S4b supports the conclusion that Figure S4b has a much better minimum hypercube SNR and does not result in any major change to the input data characteristics.

1.4. Anomaly Detection Results Normalization

Zhao *et al.* [9] used quantile normalization to normalize different types of genes. In our case, we also have diverse output values representing anomalies from different AD algorithms. Quantile normalization (quantile) can convert these results to a normal distribution without losing meaning.

Figure S5 shows how quantile normalization transformed the irregular results from KIFD into a normal distribution. Using anomaly detection scores from multiple algorithms, we have used quantile normalization on the algorithms' results and converted them to a normal distribution with the same value range, reducing imbalance without losing any information. It helped compare multiple ML models. Random Forest inherently uses information gain or Gini coefficient [10], which is not negatively impacted by scaling.

2. Supplement II

In this section, we visualize and report the performance of our Greedy Ensemble Hyperspectral Anomaly Detector (GE-AD) algorithm.

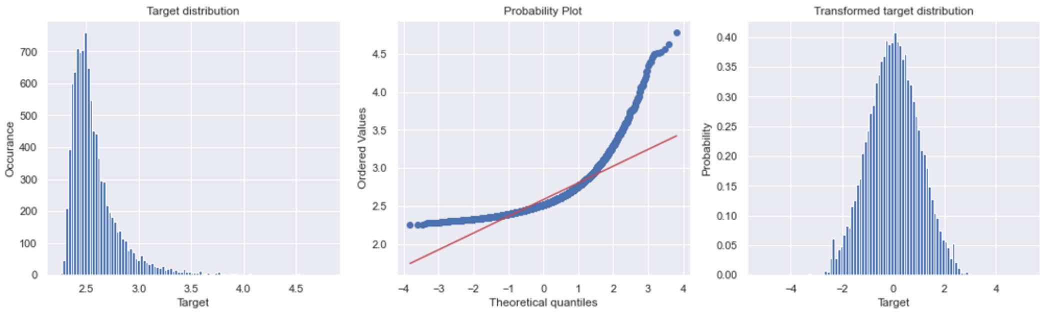

Figure S5. Irregular results from kernel isolation forest anomaly detection are transformed into a normal distribution. (a) Distribution of Original data. (b) Irregularities. (c) Transformed normal distribution.

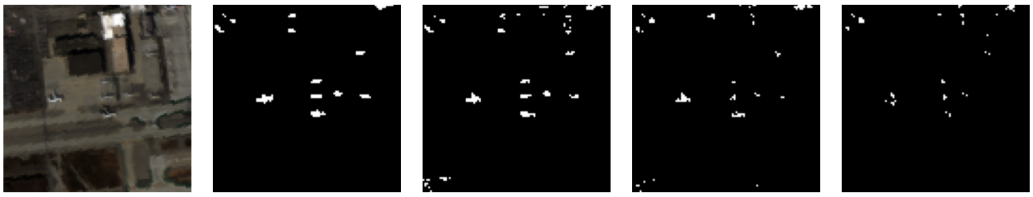

(a) **(b)** **(c)** **(d)** **(e)**

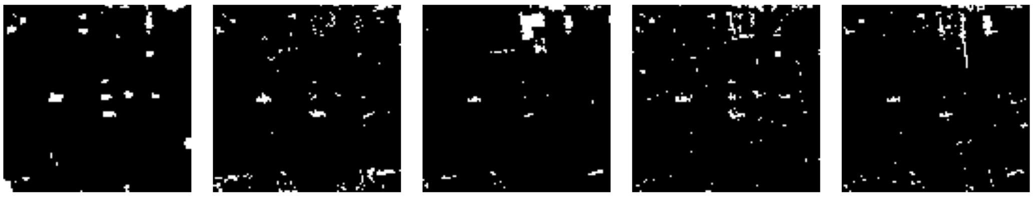

(f) **(g)** **(h)** **(i)** **(j)**

Figure S6. Visual comparison showing better performance of our proposed ensemble method (GE-AD) compared to other methods using ABU-I data. (a) RGB, (b) Ground Truth, (c) GE-AD (F1 = 0.808), (d) HUE-AD (F1 = 0.791), (e) Abundance (F1 = 0.674), (f) AED (F1 = 0.777), (g) KIFD (F1 = 0.687), (h) KRX (F1 = 0.560), (i) LSUNRSORAD (F1 = 0.693), (j) FCBAD (F1 = 0.608).

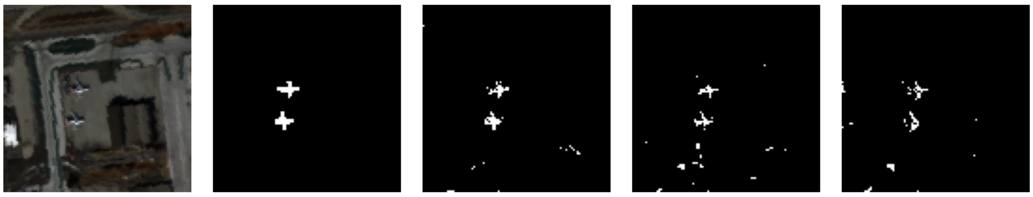

(a) **(b)** **(c)** **(d)** **(e)**

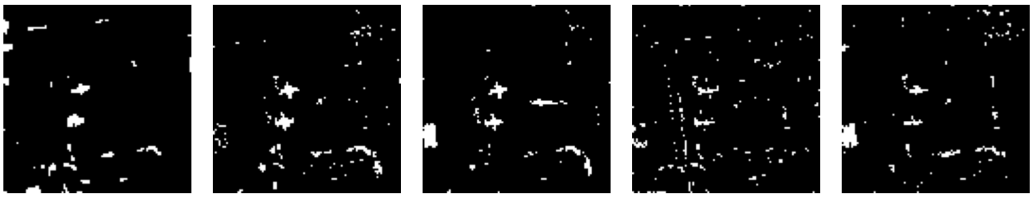

(f) **(g)** **(h)** **(i)** **(j)**

Figure S7. Visual comparison showing better performance of our proposed ensemble method (GE-AD) compared to other methods using ABU-II data. (a) RGB, (b) Ground Truth, (c) GE-AD (F1 = 0.789), (d) HUE-AD (F1 = 0.765), (e) Abundance (F1 = 0.746), (f) AED (F1 = 0.648), (g) KIFD (F1 = 0.680), (h) KRX (F1 = 0.659), (i) LSUNRSORAD (F1 = 0.606), (j) FCBAD (F1 = 0.577).

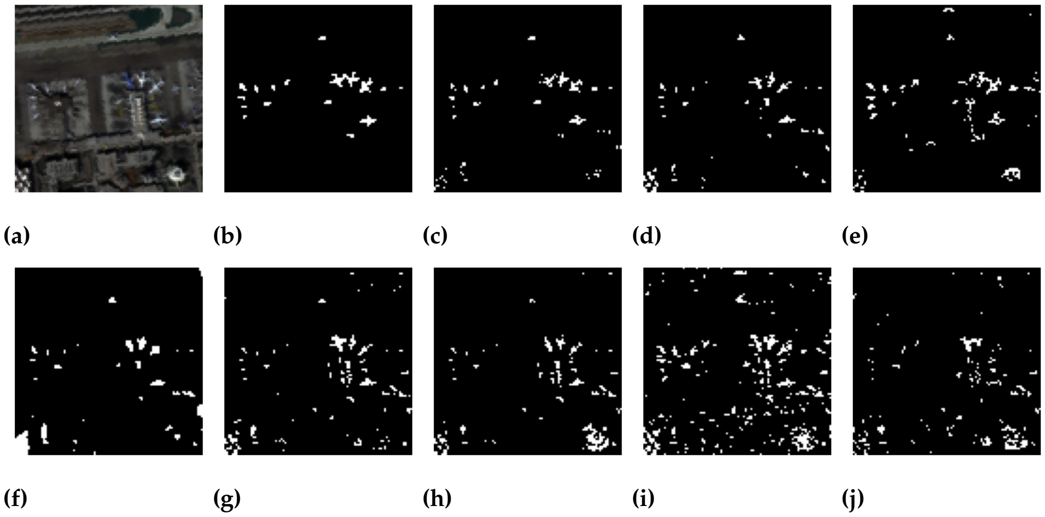

Figure S8. Visual comparison showing better performance of our proposed ensemble method (GE-AD) compared to other methods using ABU-III data. (a) RGB, (b) Ground Truth, (c) GE-AD (F1 = 0.784), (d) HUE-AD (F1 = 0.762), (e) Abundance (F1 = 0.760), (f) AED (F1 = 0.706), (g) KIFD (F1 = 0.708), (h) KRX (F1 = 0.673), (i) LSUNRSORAD (F1 = 0.660), (j) FCBAD (F1 = 0.618).

2.1. Evaluate public benchmark dataset: ABU

The GE-AD model-1 produces fewer false positives and creates a clear detection map with all the targets at least partially detected, as shown in Figures S6, S7, and S8 from the ABU dataset.

2.1.1. Investigate generalization: San Diego Airport

We also tested our GE-AD model-1 using 100 % of the San Diego dataset to evaluate the performance. Figure S9 shows the San Diego Airport dataset results. These scores are higher in San Diego-01 than the input methods used in the ensemble.

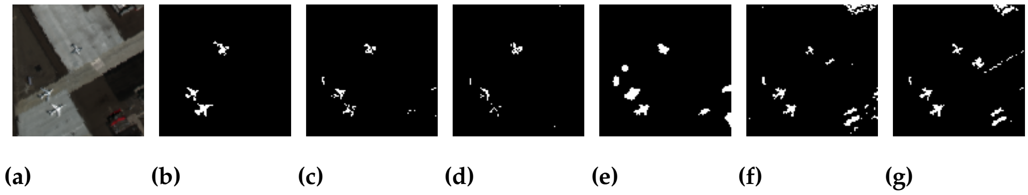

Figure S9. The visualization shows the generalization of our proposed ensemble method (GE-AD) trained on the ABU Dataset compared to other methods using San Diego-01 data. (a) RGB, (b) Ground Truth, (c) GE-AD (F1 = 0.822), (d) Abundance (F1 = 0.782), (e) AED (F1 = 0.748), (f) FCBAD (F1 = 0.691), (g) KRX (F1 = 0.722).

2.2. Evaluate public benchmark dataset: Salinas

We trained a separate GE-AD model using the Salinas dataset with seed 529 and achieved a training F1-macro score of 0.98 and a test F1-macro score of 0.98, a higher F1-macro score than any methods used in the ensemble. This model produced fewer false positives and created a clear detection map with all targets partially detected, as shown in Figure S10.

2.3. Evaluate public benchmark dataset: San Diego Airport

We trained a separate GE-AD model using the San Diego airport dataset with seed 306 and achieved a training F1-macro score of 0.86 and a test F1-macro score of 0.89, a higher F1-macro score than any methods used in the ensemble. This model produced fewer false positives and created a clear detection map with all the targets at least partially detected, as shown in Figures S11 and S12.

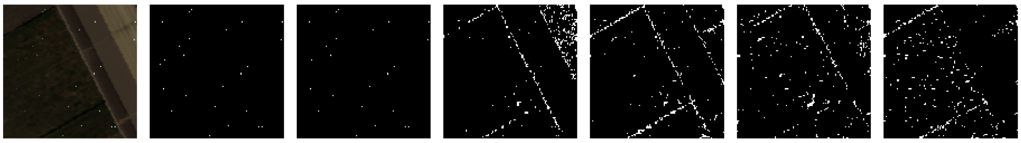

(a) **(b)** **(c)** **(d)** **(e)** **(f)** **(g)**

Figure S10. Visual comparison showing better performance of our proposed ensemble method (GE-AD) compared to other methods using Salinas data. (a) RGB, (b) Ground Truth, (c) GE-AD (F1 = 0.980), (d) CSD (F1 = 0.540), (e) FCBAD (F1 = 0.534), (f) KIFD (F1 = 0.545), (g) LSUNRSORAD (F1 = 0.551).

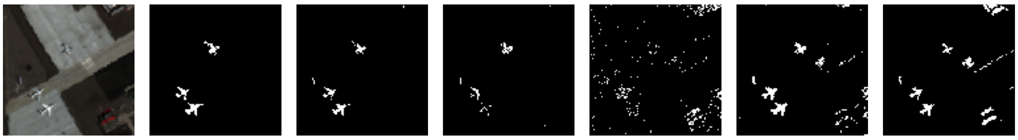

(a) **(b)** **(c)** **(d)** **(e)** **(f)** **(g)**

Figure S11. Visual comparison showing better performance of our proposed ensemble method (GE-AD) compared to other methods using San Diego-01 data. (a) RGB, (b) Ground Truth, (c) GE-AD (F1 = 0.857), (d) Abundance (F1 = 0.782), (e) GMRX (F1 = 0.553), (f) KIFD (F1 = 0.775), (g) KRX (F1 = 0.699).

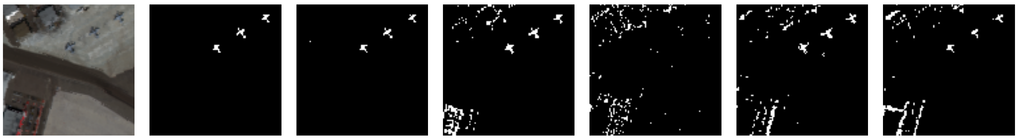

(a) **(b)** **(c)** **(d)** **(e)** **(f)** **(g)**

Figure S12. Visual comparison showing better performance of our proposed ensemble method (GE-AD) compared to other methods using San Diego-02 data. (a) RGB, (b) Ground Truth, (c) GE-AD (F1 = 0.948), (d) Abundance (F1 = 0.645), (e) GMRX (F1 = 0.496), (f) KIFD (F1 = 0.644), (g) KRX (F1 = 0.630).

2.4. *Evaluate private dataset: Arizona*

We trained a separate GE-AD model using the Arizona dataset with seed 319 and achieved a training F1-macro score of 0.81 and a test F1-macro score of 0.86, a higher F1-macro score than any methods used in the ensemble. This model produced fewer false positives and created a clear detection map with all the targets at least partially detected, as shown in Figures S13, S14, S15, and S16 from the Arizona dataset.

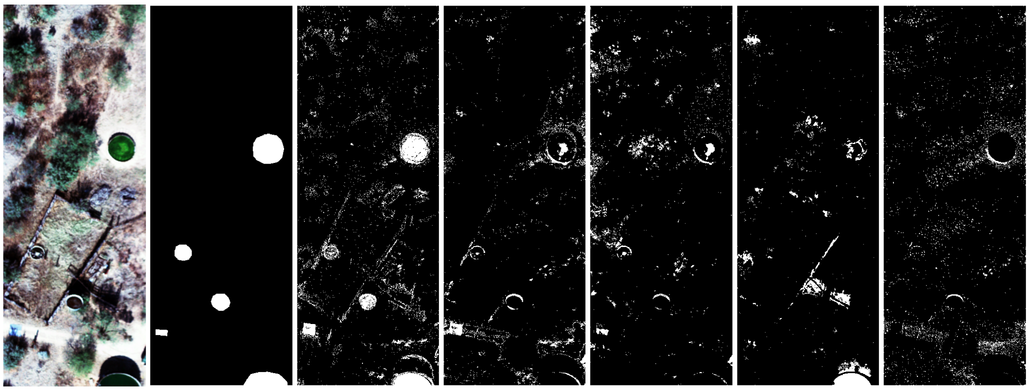

(a) **(b)** **(c)** **(d)** **(e)** **(f)** **(g)**

Figure S13. Visual comparison showing better performance of our proposed ensemble method (GE-AD) compared to other methods using Arizona image-I data. (a) RGB, (b) Ground Truth, (c) GE-AD (F1 = 0.744), (d) FCBAD (F1 = 0.590), (e) KIFD (F1 = 0.574), (f) KRX (F1 = 0.633), (g) LSUNRSORAD (F1 = 0.522).

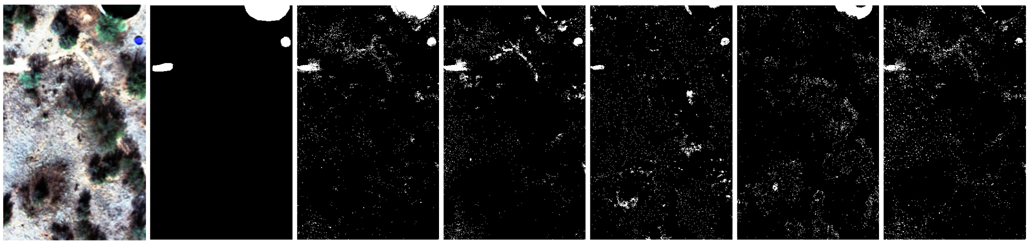

(a) **(b)** **(c)** **(d)** **(e)** **(f)** **(g)**

Figure S14. Visual comparison showing better performance of our proposed ensemble method (GE-AD) compared to other methods using Arizona image-II data. (a) RGB, (b) Ground Truth, (c) GE-AD (F1 = 0.766), (d) FCBAD (F1 = 0.613), (e) KIFD (F1 = 0.565), (f) KRX (F1 = 0.680), (g) LSUNRSORAD (F1 = 0.571).

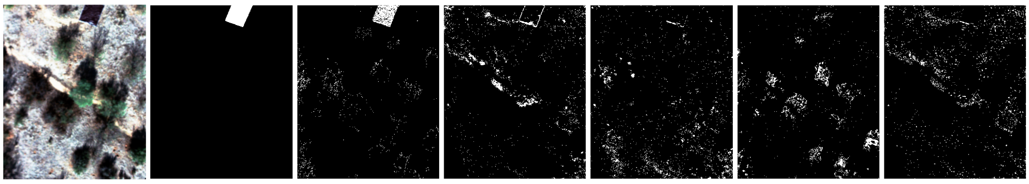

(a) **(b)** **(c)** **(d)** **(e)** **(f)** **(g)**

Figure S15. Visual comparison showing better performance of our proposed ensemble method (GE-AD) compared to other methods using Arizona image-IV data. (a) RGB, (b) Ground Truth, (c) GE-AD (F1 = 0.791), (d) FCBAD (F1 = 0.547), (e) KIFD (F1 = 0.507), (f) KRX (F1 = 0.512), (g) LSUNRSORAD (F1 = 0.498).

3. Supplement III

In this section, we discuss the details and results of our first ablation study, where we considered the impact of noise on the ensemble.

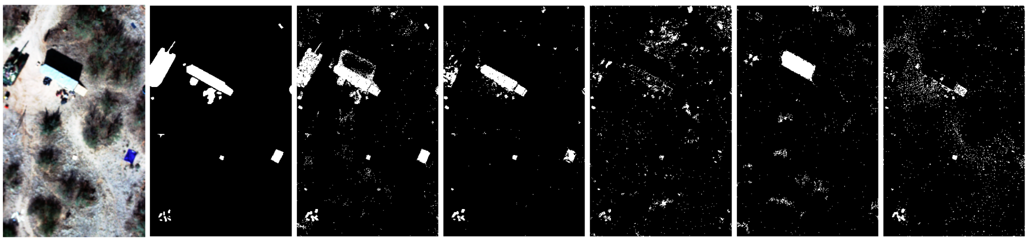

(a) **(b)** **(c)** **(d)** **(e)** **(f)** **(g)**

Figure S16. Visual comparison showing better performance of our proposed ensemble method (GE-AD) compared to other methods using Arizona image-V data. (a) RGB, (b) Ground Truth, (c) GE-AD (F1 = 0.859), (d) FCBAD (F1 = 0.809), (e) KIFD (F1 = 0.582), (f) KRX (F1 = 0.502), (g) LSUNRSORAD (F1 = 0.576).

Table S3. The impact of noise on our proposed ensemble method (GE-AD) using F1-macro on the ABU dataset.

Added noise	Methods	ABU-I	ABU-II	ABU-III	ABU-IV
0 % added noise	GE-AD	0.819	0.797	0.783	0.79
	Abundance	0.643	0.759	0.746	0.714
	AED	0.769	0.643	0.714	0.602
	FCBAD	0.622	0.6	0.629	0.624
	KRX	0.559	0.66	0.691	0.592
5 % increase in STD of pixel values	GE-AD	0.725	0.794	0.757	0.706
	Abundance	0.685	0.676	0.743	0.546
	AED	0.692	0.713	0.69	0.645
	FCBAD	0.601	0.597	0.61	0.644
	KRX	0.564	0.66	0.689	0.593
15 % increase in STD of pixel values	GE-AD	0.706	0.615	0.749	0.717
	abundance	0.645	0.539	0.722	0.7
	aed	0.703	0.713	0.686	0.649
	fcbad	0.571	0.585	0.596	0.624
	krx	0.57	0.659	0.686	0.592

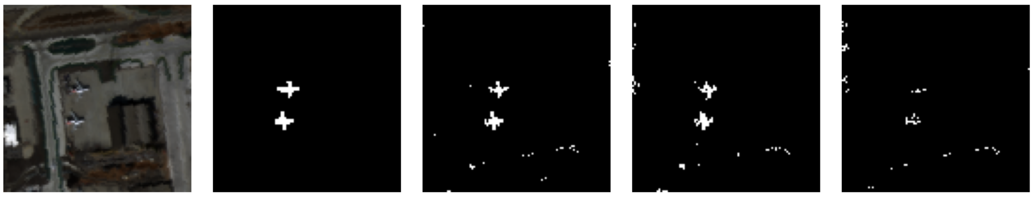

(a) **(b)** **(c)** **(d)** **(e)**

Figure S17. Visual comparison showing qualitative performance of our proposed ensemble method (GE-AD) in the presence of added noise using ABU-II data. (a) RGB, (b) Ground Truth, (c) GE-AD on original data (F1 = 0.797), (d) GE-AD on 5 % increased STD data (F1 = 0.794), (e) GE-AD on 15 % increased STD data (F1 = 0.615)

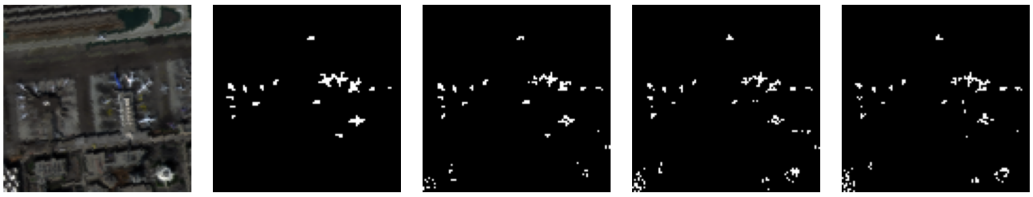

(a) **(b)** **(c)** **(d)** **(e)**

Figure S18. Visual comparison showing qualitative performance of our proposed ensemble method (GE-AD) in the presence of added noise using ABU-III data. (a) RGB, (b) Ground Truth, (c) GE-AD on original data (F1 = 0.783), (d) GE-AD on 5 % increased STD data (F1 = 0.757), (e) GE-AD on 15 % increased STD data (F1 = 0.749)

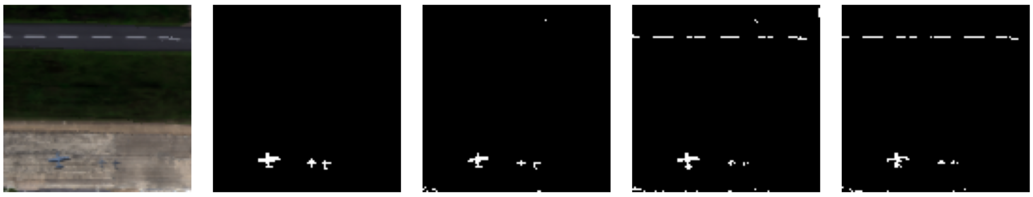

(a) **(b)** **(c)** **(d)** **(e)**

Figure S19. Visual comparison showing qualitative performance of our proposed ensemble method (GE-AD) in the presence of added noise using ABU-IV data. (a) RGB, (b) Ground Truth, (c) GE-AD on original data (F1 = 0.790), (d) GE-AD on 5 % increased STD data (F1 = 0.706), (e) GE-AD on 15 % increased STD data (F1 = 0.717)

Funding: This research was sponsored by the Army Research Laboratory and was accomplished under Cooperative Agreement Number W911NF-21-2-0294 with E. L. Jacobs as PI. The views and conclusions contained in this document are those of the authors and should not be interpreted as representing the official policies, either expressed or implied, of the Army Research Office or the U.S. Government. The U.S. Government is authorized to reproduce and distribute reprints for Government purposes notwithstanding any copyright notation herein.

Funding: This material is based upon work supported by the National Science Foundation while Lan Wang was serving at the National Science Foundation. Any opinion, findings, and conclusions or recommendations expressed in this material are those of the author(s) and do not necessarily reflect the views of the National Science Foundation.

References

1. Watson, T.P.; McKenzie, K.; Robinson, A.; Renshaw, K.; Driggers, R.; Jacobs, E.L.; Conroy, J. Evaluation of aerial real-time RX anomaly detection. In Proceedings of the Algorithms, Technologies, and Applications for Multispectral and Hyperspectral Imaging XXIX. SPIE, 2023, Vol. 12519, pp. 254–260.
2. Keshava, N.; Mustard, J.F. Spectral unmixing. *IEEE signal processing magazine* **2002**, *19*, 44–57.
3. Younis, M.S.; Hossain, M.; Robinson, A.L.; Wang, L.; Preza, C. Hyperspectral unmixing-based anomaly detection. In Proceedings of the Computational Imaging VII. SPIE, 2023, Vol. 12523, p. 1252302.
4. Chang, C.I.; Du, Q. Estimation of number of spectrally distinct signal sources in hyperspectral imagery. *IEEE Transactions on Geoscience and Remote Sensing* **2004**, *42*, 608–619. https://doi.org/10.1109/TGRS.2003.819189.
5. Winter, M.E. N-FINDR: an algorithm for fast autonomous spectral end-member determination in hyperspectral data. In Proceedings of the Imaging Spectrometry V; Descour, M.R.; Shen, S.S., Eds. International Society for Optics and Photonics, SPIE, 1999, Vol. 3753, pp. 266 – 275. https://doi.org/10.1117/12.366289.
6. Chang, C.I. An information-theoretic approach to spectral variability, similarity, and discrimination for hyperspectral image analysis. *IEEE Transactions on Information Theory* **2000**, *46*, 1927–1932. https://doi.org/10.1109/18.857802.
7. Du, Q.; Yang, H. Similarity-based unsupervised band selection for hyperspectral image analysis. *IEEE geoscience and remote sensing letters* **2008**, *5*, 564–568.
8. Lyngdoh, R.B.; Sahadevan, A.S.; Ahmad, T.; Rathore, P.S.; Mishra, M.; Gupta, P.K.; Misra, A. Avhyas: A free and open source qgis plugin for advanced hyperspectral image analysis. In Proceedings of the 2021 International Conference on Emerging Techniques in Computational Intelligence (ICETCI). IEEE, 2021, pp. 71–76.
9. Zhao, Y.; Wong, L.; Goh, W.W.B. How to do quantile normalization correctly for gene expression data analyses. *Scientific reports* **2020**, *10*, 15534.

10. Dorfman, R. A formula for the Gini coefficient. *The review of economics and statistics* **1979**, pp. 146–149.

Disclaimer/Publisher's Note: The statements, opinions and data contained in all publications are solely those of the individual author(s) and contributor(s) and not of MDPI and/or the editor(s). MDPI and/or the editor(s) disclaim responsibility for any injury to people or property resulting from any ideas, methods, instructions or products referred to in the content.
